# Anti-SARS-CoV-2 seroprevalence in King County, WA—Cross-sectional survey, August 2020

**DOI:** 10.1371/journal.pone.0272783

**Published:** 2022-08-22

**Authors:** Karen D. Cowgill, Elena A. Erosheva, Adam Elder, Ljubomir Miljacic, Susan Buskin, Jeffrey S. Duchin

**Affiliations:** 1 Department of Global Health, School of Public Health, University of Washington, Seattle, Washington, United States of America; 2 Department of Statistics, School of Social Work, and the Center for Statistics and the Social Sciences, University of Washington, Seattle, Washington, United States of America; 3 Department of Biostatistics, School of Public Health, University of Washington, Seattle, Washington, United States of America; 4 The Mountain-Whisper-Light, Seattle, Washington, United States of America; 5 Public Health–Seattle & King County, Seattle, Washington, United States of America; 6 Department of Epidemiology, School of Public Health, University of Washington, Seattle, Washington, United States of America; 7 Department of Medicine, Division of Allergy & Infectious Diseases, School of Medicine, University of Washington, Seattle, Washington, United States of America; Food and Drug Administration, UNITED STATES

## Abstract

We conducted a seroprevalence survey to estimate the true number of infections with SARS-CoV-2, the virus that causes COVID-19, in King County as of August 2020 by measuring the proportion of residents from who had antibodies against the virus. Participants from 727 households took part in a cross-sectional address-based household survey with random and non-random samples and provided dried blood spots that were tested for total antibody against the viral nucleocapsid protein, with confirmatory testing for immunoglobulin G against the spike protein. The data were weighted to match King County’s population based on sex, age group, income, race, and Hispanic status. After weighting and accounting for the accuracy of the tests, our best overall estimate of anti-SARS-CoV-2 seroprevalence in King County as of August 2020 is 3.9% (95% confidence interval (CI) 2.4%-6.0%) with an effective sample size of 589. Comparing seroprevalence with positive test reports, our survey suggests that viral testing underestimated incidence by a factor of about five and suggests that the proportion of cases that were serious (based on hospitalization) or fatal was 2.4% and 0.8%, respectively. Prevalence varied by subgroup; households reporting incomes at or below $100,000 in 2019 had nearly five times higher estimated antibody prevalence than those with incomes above $100,000. Those reporting non-White/non-Asian race had roughly seven times higher estimated antibody prevalence than those reporting White race. This survey was noteworthy for including people of all ages; among all age groups, the weighted estimate of prevalence was highest in older teens and young adults and lowest in young children, although these differences were not statistically significant.

## Introduction

Severe acute respiratory syndrome coronavirus 2 (SARS-CoV-2), the virus that causes COVID-19, has been circulating in western Washington state since at least late January 2020 [1]. Polymerase chain reaction (PCR) testing to detect active infection was limited in the early months of the outbreak, and access to and use of testing was uneven around King County, with lower rates of testing among racial and ethnic groups and in the southern part of the county through July 2020 [2]. The true number of infections in King County residents was unknown; mathematical models based on detected cases of active infection along with other sources of data had produced estimates that are 10 or more times higher than reported cases in other parts of the United States (US) [3, 4]. This seroprevalence survey aimed to help understand the true number of infections in King County as of August 2020 by measuring the proportion of residents who had evidence of past infection with SARS-CoV-2.

### Objectives

The objectives of this survey were to:

1. Estimate the prevalence of antibody to SARS-CoV-2 among the 2.26 million residents of King County, Washington and
2. Explore which groups bear a higher burden of disease by estimating cumulative incidence in subgroups of the population stratified by age, sex, income, and race/ethnicity and
3. Assess virulence by calculating the symptomatic proportion of cases and ratio of severe disease among those with evidence of past infection with SARS-CoV-2.

## Methods

### Survey design

A cross-sectional address-based household survey with random and non-random (convenience) samples to measure anti-SARS-CoV-2 prevalence at a single time.

### Sample size

We anticipated a seroprevalence of about 2.5%, so we aimed to complete surveys from 800 households with an average of 2.5 members to obtain a reasonably precise estimate.

### Sampling methodology

Sampling and recruitment methods for the random and non-random samples differed. The random sample was a stratified probability sample of 5,000 households with an oversampling of households from selected census block groups (CBGs) with a high density (30% or more) of households where at least one member identified as Black/African American or, separately, Hispanic/Latinx. This random sample was supplemented by a non-random sample to aim for a target of at least 50 households with at least one member who identified as Native Hawaiian/Pacific Islander and 50 households with at least one member who identified as American Indian/Alaska Native.

For the random samples, we contracted with a survey firm (Marketing Systems Group (MSG), www.M-S-G.com) to pull an address-based sample (ABS) that met these requirements and then sent postcards in English and Spanish inviting 2,500 randomly sampled households from targeted CBGs for African American and Hispanic households (1,250 each) and 2,500 randomly sampled households from the remaining CBGs to participate in our study; the postcards also had short messages in Korean, Simplified Chinese, Amharic, Somali, and Vietnamese, languages identified by the Public Health Seattle & King County (PHSKC) Language Liaison to be the most relevant for recruiting a representative sample. To recruit additional non-randomly sampled households from underrepresented groups, we enlisted the help of community-based organizations (CBOs).

### Survey location

Households in King County, Washington were eligible for the survey. King County includes the city of Seattle and covers an area of 2,307 square miles.

For the address-based random sample, five fixed specimen collection events were held at different locations between August 8^th^ and 15^th^ andmobile teams wearing personal protective equipment collected specimens outdoors at the homes of participants not able to attend the specimen collection events.

For the non-random sample, there were two fixed-site events on August 26^th^ and 29^th^.

### Survey population

King County’s total population in 2020 was about 2.26 million [5], nearly one quarter of Washington State’s population. The address-based sample included residents living at selected non-institutional addresses; households of any size, including single people, were eligible to participate. Landline and/or cell phone numbers were appended to just over half the 5,000 postal addresses, and 22% had email addresses as well. We sent text messages and emails inviting participation. Anyone who responded and lived in King County was eligible to participate. For the non-address-based sample, any resident of King County was eligible to participate.

### Community outreach and engagement

The survey page on the PHSKC website had information about the survey, a link to the online questionnaire, and Frequently Asked Questions in English, Spanish, Amharic, Simplified and Traditional Chinese, Korean, and Vietnamese. Survey organizers reached out to organizations representing individual American Indian tribes in King County as well as to the Urban Indian Health Initiative and the Seattle Indian Health Board in an effort to oversample this population [6] and the Pacific Islander community. The survey was featured in a weekly Spanish community livestream conversation on COVID-19 and in email information and/or presentations to community health boards, community navigators, participants in the weekly PHSKC Community Health Services COVID-19 and Racial Equity call, and members of the PHSKC Community Mitigation and Response Group. PHSKC Communications advertised the non-random-sample specimen-collection event on social media and via the PHSKC Public Health Insider blog (www.publichealthinsider.com).

### Data collection

The questionnaire requested information on demographics, COVID-19 symptoms and illness episodes, medical history, and social risk factors for COVID-19 illness and was available online in English and Spanish. It was based on a survey instrument from the Centers for Disease Control and Prevention’s COVID-19 Community Seroepidemiological Investigation [7] and on the first version of the Social Risk Factors for COVID-19 Exposure Survey, posted May 21, 2020 [8].

**Fig 1:**
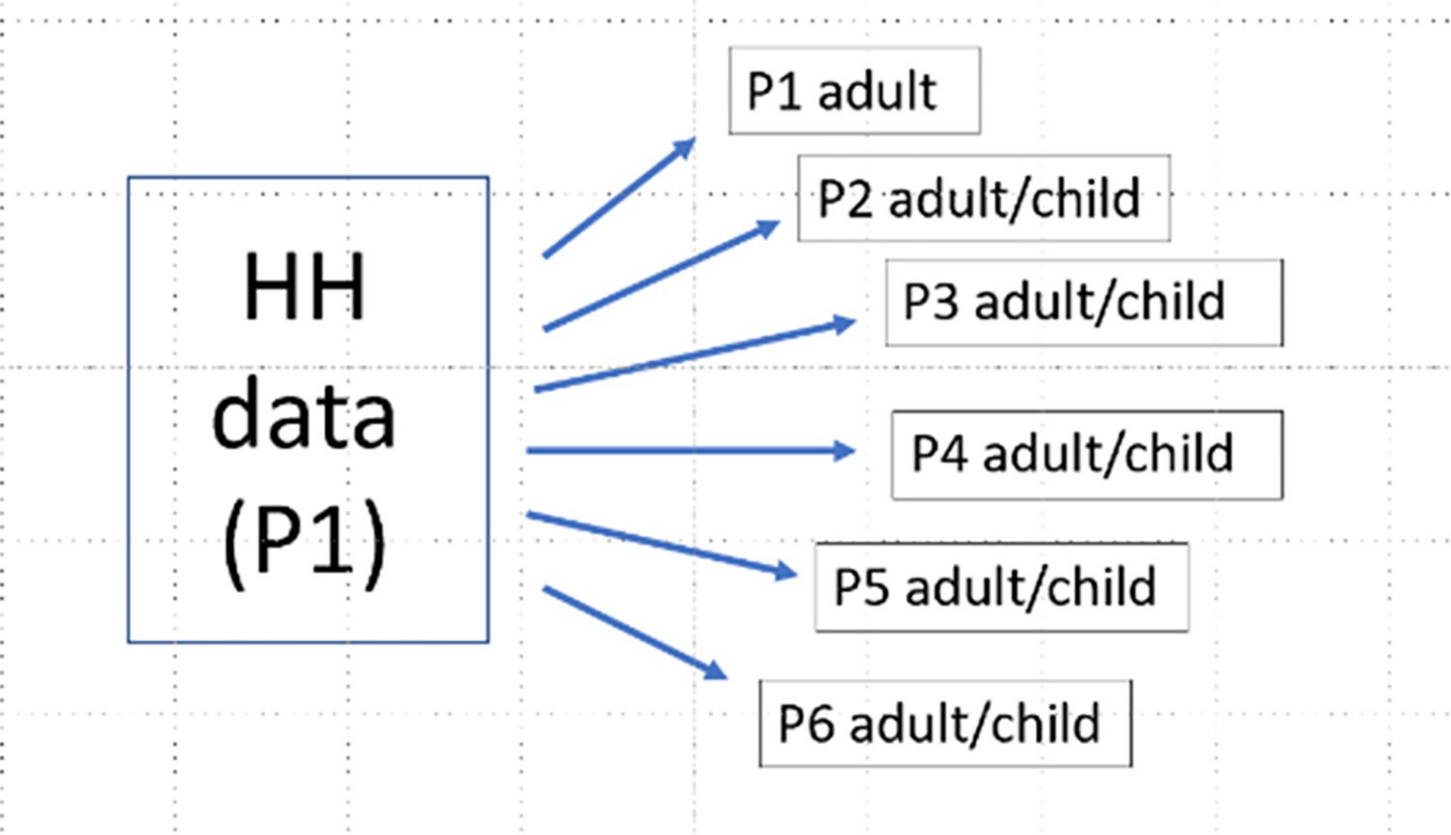
Database architecture, King County, WA COVID-19 antibody testing survey, August 2020.

The questionnaire was designed to allow for responses from up to six members of participating households (HH), with the first adult member (Person 1, or P1) completing an initial HH questionnaire and an individual questionnaire followed by up to five other adult or child members (P2-P6) completing individual questionnaires (**Fig 1**). Each consenting adult HH member was to complete an individual questionnaire or have one completed for them if they were unable to complete it themselves. Adolescents (ages 13–17) who gave assent and whose parents or guardians consented were to either complete an individual questionnaire themselves or have one completed for them, and parents or guardians who consented were to complete one for their children younger than 13.

The survey questionnaire was entered into REDCap (Institute of Translational Health Sciences, Seattle, WA, USA), with a link via the PHSKC website for participants to complete the survey on a mobile device or computer. For participants who were not able to or who chose not to complete the survey themselves, there was a back-up option to complete it by phone. For the address-based sample, the first adult member of the household completed a household questionnaire and scheduled an appointment at one of the fixed sites or requested a visit from a mobile team for collection of blood specimens from consenting/assenting household members or children. The process was the same for the non-random sample, except that instead of requesting appointments, participants were invited to attend one of the two fixed sites on specified dates, and no home visits were offered.

Phone interpretation services were available for those who spoke a language other than English or Spanish.

### Data management

All data generated from this survey were managed and stored according to established data management principles. Confidentiality and security methods included confirming that all project staff and PHRC volunteers had been trained in protecting participant confidentiality and had signed a workforce agreement laying out the best practices for security and confidentiality and the consequences for data breaches. All electronic data are stored in password-protected locations and stored on a HIPAA-compliant site at the University of Washington’s Institute of Translational Health Sciences (www.iths.org).

### Ethical considerations

This survey was considered a public health activity and as such was not required to undergo institutional review board review. Nonetheless, human subjects protections were implemented, including consent from all adult participants and assent from minors, the right to refuse or withdraw from participation, and protection of confidential health information. Each household that completed the survey (including providing a blood specimen) received a $20 grocery gift card to acknowledge the time and inconvenience of participating.

### Specimen collection and storage

Survey staff collected blood specimens from participants via fingerstick. Four to five blood spots (a total of 240–350 microliters) were collected on a filter-paper card and dried and shipped overnight to the testing laboratory, Molecular Testing Labs (MTL), in Vancouver, Washington. Some 142, or 10%, of the dried blood spot specimens collected could not be tested by the lab because the quantity of blood collected was insufficient. We were able to re-contact and collect second specimens from 63 people, but there were 79 participants whose specimens could not be tested.

### Specimen testing

Upon receipt at MTL, the DBS were eluted and tested via a validated in-house adaptation of the Bio-Rad Platelia qualitative ELISA for total antibody (IgG, IgA, IgM) against the nucleocapsid protein of SARS-CoV-2 [9]. All samples that were positive by this first assay were then tested using a validated in-house adaptation of the EuroImmun qualitative ELISA for IgG against the spike protein of SARS-CoV-2 [10]. The unweighted count of participants positive for antibody to SARS-CoV-2 was 33; an additional nine were positive only on the first assay.

### Results sharing and use

Individual participants received a printed report of their test results by postal mail. The lab notified Washington State Department of Health of positive antibody test results, including identifying information as required by Washington Administrative Code.

### Data analysis

We extracted demographic variables for each responding household member and sent a cleaned, de-identified version of this dataset to MSG, where a weighted sequential hot-deck procedure was used to impute missing values for the demographic variables (age group, race, ethnicity, education, income, and household size). The random and non-random samples were combined for the analysis. MSG used the dual frame where the random (address-based) sample and the non-random sample were assigned initial uniform weights to generate adjusted sampling weights for each household and individual participant via raking. Specifically, raking was done to King County’s population data on sex, age group, income, race, and Hispanic status distributions. The weights adjusted for non-response and under-coverage.

The weighted data were used to obtain seroprevalence estimates. Only individuals who tested positive on both tests were counted as positive. This combination assumes that the tests are independent conditional on each individual’s true status and allows us to treat this sequential testing procedure as a single test (**Table 1**). The seroprevalence estimates were adjusted for missingness on the response variable–as noted above, 79 participants’ blood specimens were insufficient for testing–and the combined sensitivity and specificity of the sequential antibody assays. In addition, statistical adjustments were considered to correct for the fact that individuals in the non-random sample were more likely to have had a COVID-19-like illness; however, these adjustments are not included in our best seroprevalence estimate due to the lack of suitable population benchmarks. Detailed statistical methods are presented in The Prevalence estimation report (S2 File).

**Table 1:** Performance of serial antibody assays, King County, Washington anti-SARS-CoV-2 seroprevalence survey, August 2020.

Test	Negative percent agreement (specificity)	Positive percent agreement (sensitivity)
Bio-Rad Ab test [9]	99.56%	92.16%
EuroImmun ELISA (IgG) [10]	100%	90%
Sequential Test [11]	100%	82.94%

We also tested for between-group difference across four of the demographic variables (race, sex, income, and age group; as noted below, there were too many missing values to look at ethnicity). Because these between-group differences tests contain multiple hypotheses (one for each combination of levels within a category, for each category), we adjusted the p-values to control type-one error.

For these comparisons, we followed the guidance found in chapter 8.5 of Cox and Donnelly’s Principles of Applied Statistics [12] and in a publication by Bender and Lange [13]. We considered pairwise comparisons between different levels of a categorical variable as part of a single question. However, we treated hypotheses concerned with different categorical variables as distinct from one another. As an example, we considered the comparison between Blacks and Whites and the comparison between Blacks and Asians as part of a single question but considered the comparison between Blacks and Asians separate from the comparison between males and females. Thus, we controlled for the family-wise error rate using a Bonferroni adjustment for pairwise comparisons between different groups within each category but did not adjust for the number of categorical variables for which we were making between-category comparisons (in our case, four). While it is common to use an alpha level of 0.05 for testing procedures, here we followed the guidance of Benjamin, et al [14] and set our alpha level to be 0.005 to reduce the risk of incorrectly "discovering" a new result.

## Results

Our best overall estimate of anti-SARS-CoV-2 seroprevalence in King County as of August 2020 is 3.9% (95% confidence interval (CI) 2.4%-6.0%). Based on our analyses comparing the random and the non-random samples, it is likely that our best estimates are slightly biased upward. See The Prevalence estimation report (S2 File) for details.

Subgroup analysis by race, sex, income, and age group are calculated using the adjustments described in The Prevalence estimation report (S2 File) and are based on a single imputation of missing values. In the original dataset, the proportion of missing values among key demographic variables ranged from 0–46% (**Table 2**) due to errors in the design of the online questionnaire that allowed respondents to skip some demographic information questions that were intended to be required.

**Table 2:** Percent of missing values, King County, Washington anti-SARS-CoV-2 seroprevalence survey, August 2020 (n = 1,364).

Variable	Percent Missing
Sex	0*
Age Category	3.15
Income	9.09
Race	11.00
Ethnicity	45.53

*Sex was a required field for laboratory testing

During the initial survey phase through August 16^th^, 860 households from the address-based sample initiated the online questionnaire, of which 417 (48.5%) completed the questionnaire and provided a blood specimen. After August 16^th^, 844 households in the non-random sample initiated the questionnaire, of which 310 (36.7%) completed it and provided a blood specimen. Overall, there was a total of 727 participating households with an average of 1.88 participating individuals per household. There were 3,163 individual questionnaire starts online, of which 1,364 (43.1%) are associated with blood specimens. Taking into account missingness and household clustering, the effective sample size was 589.

The number of participants identifying as Native Hawaiian or Other Pacific Islanders and American Indian or Alaska Natives and the number of participants for whom information about Hispanic ethnicity was available were too low to generate separate estimates for these groups. Thus, people of Hispanic ethnicity are represented only by race, and those who identified as Native Hawaiian or Pacific Islander and American Indian or Alaska Native are subsumed in the ‘Other’ category. The unweighted proportion of participants by race after imputation compared to the proportions in King County are shown in **Table 3**.

**Table 3:** Proportion of participants by race, King County, Washington anti-SARS-CoV-2 seroprevalence survey, August 2020.

Race	King County 2020	Unweighted Survey Sample
White	66.1%	69.9%
Black or African-American	7.2%	6.0%
American Indian or Alaska Native	1.0%	--*
Asian	19.3%	20.5%
Native Hawaiian or Other Pacific Islander	0.9%	--*
Two or more races	5.5%	--*
Other	-	3.7%

*numbers in these categories were small and were collapsed into the ‘Other’ category before imputation of missing values

**Fig 2:**
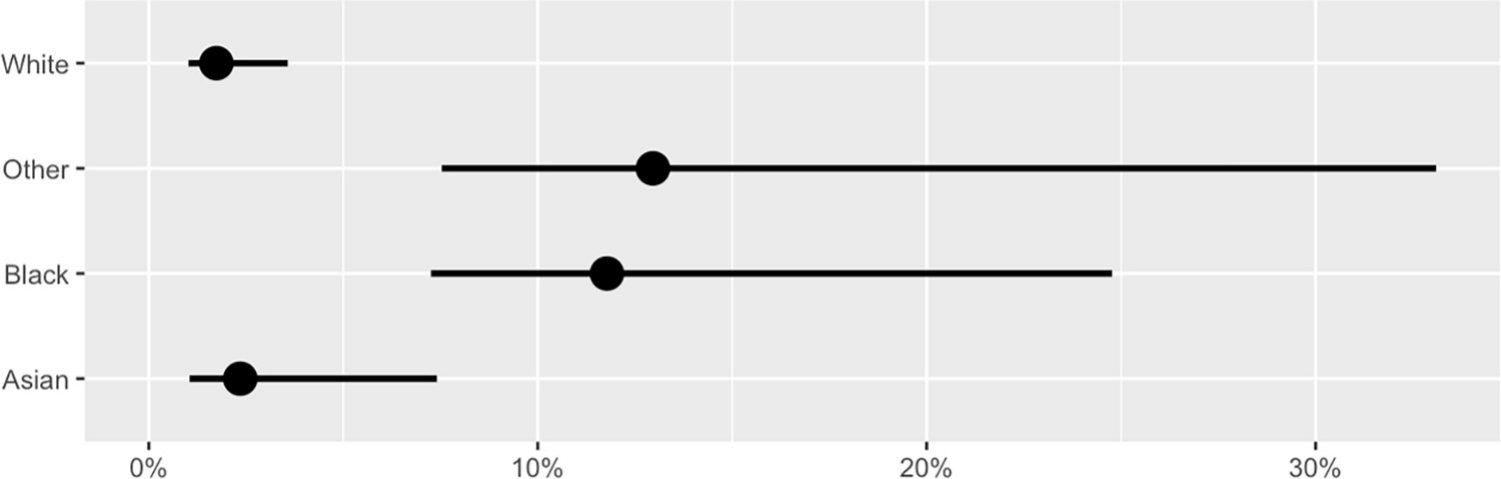
Estimated anti-SARS-CoV-2 seroprevalence across race group in King County in early-to-mid-August 2020. Points indicate estimates and lines indicate 95% confidence intervals.

**Fig 3:**
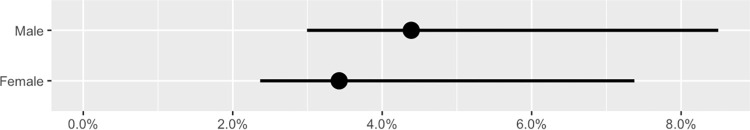
**Estimated anti-SARS-CoV-2 seroprevalence across sex in King County in early-to-mid-August 2020.** Points indicate estimates and lines indicate 95% confidence intervals.

**Fig 4:**
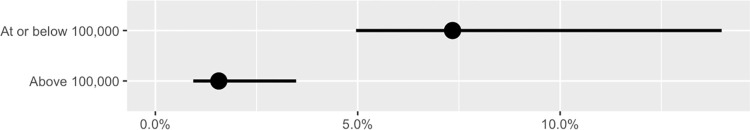
Estimated anti-SARS-CoV-2 seroprevalence across income categories in King County in early-to-mid-August 2020. Income categories in United States dollars. Points indicate estimates and lines indicate 95% confidence intervals.

**Fig 5:**
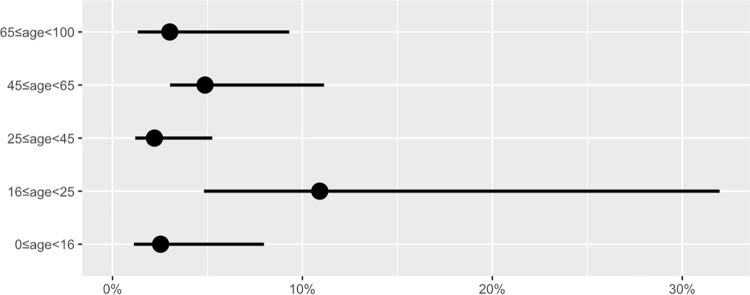
Estimated anti-SARS-CoV-2 seroprevalence across age group in King County in early-to-mid-August 2020. Age in years. Points indicate estimates and lines indicate 95% confidence intervals.

Prevalence estimates and the associated 95% confidence intervals for participants identifying as White, Black, and Asian are shown in **Table 4** and in the accompanying plot (**Fig 2**). Prevalence point estimates were approximately seven times higher in those identifying as Black and Other than in those identifying as White, though due to the small sample sizes in these groups, the confidence intervals are wide. Estimated point prevalence was slightly higher in those reporting male sex than in females (**Table 4 and Fig 3**), although more females participated. Estimated point prevalence was more than four and a half times higher in those who reported their 2019 household income was at or below $100,000 as in those with incomes above $100,000 (**Table 4 and Fig 4**). Finally, point prevalence estimates varied substantially by age group and suggest that seroprevalence was highest in the 16-25-year age group at the time of the survey (**Table 4 and Fig 5**).

**Table 4:** Weighted prevalence estimates for anti-SARS-CoV-2, King County, Washington anti-SARS-CoV-2 seroprevalence survey, August 2020.

	Unweighted Number (*number missing and imputed*)	Unweighted Percentage	Weighted Prevalence Estimate (95% CI)
**Total**	1364	100%	**3.91 (2.35–5.96)**
**Race**			
White	953 (*110*)	69.9%	**1.74 (1.02–3.57)**
Black	82 (*6*)	6.0%	**11.78 (7.25–24.77)**
Asian	279 (*25*)	20.5%	**2.35 (1.05–7.41)**
Other	50 (*9*)	3.7%	**12.96 (7.53–33.10)**
**Sex**			
Male	650 (*0*)	47.7%	**4.39 (2.99–8.50)**
Female	714 (*0*)	52.3%	**3.42 (2.37–7.38)**
**Income** ^**1**^			
At or below $100,000	585 (*57*)	42.9%	**7.34 (4.96–13.99)**
Above $100,000	779 (*67*)	57.1%	**1.57 (0.94–3.48)**
**Age Group (years)**			
65 - < 100	206 (*8*)	15.1%	**3.01 (1.32–9.31)**
45 - < 65	391 (*10*)	28.7%	**4.87 (3.03–11.14)**
25 - < 45	488 (*12*)	35.8%	**2.21 (1.19–5.25)**
16 - < 25	108 (*4*)	7.9%	**12.18 (6.10, 31.10)**
0 - <16	171 (*9*)	12.5%	**1.65 (0.29, 5.97)**

After considering all pairwise differences between groups, the following group differences were found to be statistically significant at the 0.005 level. The Bonferroni-adjusted p-values for all pairwise comparisons are reported in The Prevalence estimation report (S2 File). While the Bonferroni adjustment is conservative, all pairwise differences not found statistically significant using the Bonferroni adjustment also failed to reach significance using the less conservative procedure described by Benjamini and Yakutieli [15].

We found significant differences between people reporting Other and White and Black and White race (**Table 5)**:

**Table 5:** Tests of differences by race, King County, Washington anti-SARS-CoV-2 seroprevalence survey, August 2020.

Baseline Group	Comparison Group	Estimated Odds Ratio	Confidence Interval	Bonferroni-adjusted p-value
White	Other	8.2	(2.5, 26.9)	0.003113
White	Black	7.3	(2.6, 20.6)	0.000978

We also found statistically significant differences between those with an annual income of less than $100,000 and those with an annual income of $100,000 or more (**Table 6**).

**Table 6:** Tests of differences by income, King County, Washington anti-SARS-CoV-2 seroprevalence survey, August 2020.

Baseline Group	Comparison Group	Estimated Odds Ratio	Confidence Interval	Bonferroni-Adjusted P-value
Above $100,000	At or below $100,000	5.0	(1.9, 12.8)	0.000984

## Discussion

Our best estimate of the proportion of King County residents with antibodies against SARS-CoV-2, the virus that causes COVID-19, in August 2020 is about 4%, or 90,000 people. Based on our analyses from comparing the random and the non-random samples, it is likely that our best estimates are slightly biased upward. However, this correction is not presented in the main analysis since the extent of this bias is uncertain. Vaccines were still in clinical trials at the time of this survey and therefore none of the participants would have received a vaccine against COVID-19 unless they were participants in a clinical trial. The first antibody assay in the serial testing algorithm detected total antibody to the nucleocapsid protein, which is generated by natural infection, but not by vaccination, so we are confident that this estimate reflects natural infection and not vaccine-induced immune response.

A total of 18,060 positive test results for SARS-CoV-2 had been reported to King County through August 15^th^, 2020, the midpoint of our survey [2]. Assuming each of these represented a unique test result of a King County resident, the reported period prevalence of infection up until that date was at most 0.8% of the county’s population; our survey thus suggests that viral testing underestimated incidence by a factor of about five. There were 2,192 hospitalizations and 698 deaths through August 15^th^ [2], suggesting the proportion of cases that were serious (based on hospitalization) or fatal was 2.4% and 0.8%, respectively.

As expected, prevalence varied by subgroup. We saw that households reporting incomes at or below $100,000 in 2019 had nearly five times higher estimated antibody prevalence than those with incomes above $100,000. Data on race and ethnicity were limited but indicated that those reporting non-White/non-Asian race had roughly seven times higher estimated antibody prevalence than those reporting White race. This survey was noteworthy for including people of all ages; our data suggest that older teens and young adults in King County had higher antibody prevalence at the time of this survey than did people of other ages, and younger children had the lowest prevalence, although these group differences were not found to be statistically significant at the 0.005 significance level to qualify for “new discoveries” [15].

This was the first data-based local estimate of anti-SARS-CoV-2 seroprevalence for King County. Our estimate is somewhat higher than those from other sources generated around the same time. National commercial laboratory seroprevalence data show that in the latter half of August 2020, the seroprevalence for Washington State as a whole was estimated at 3.0% (95% CI 1.8–4.5%) [16]. Data from the 10-Site Commercial Laboratory Seroprevalence Survey show a considerably lower estimated seroprevalence for the Western Washington Region at the end of July 2020 of just 1.3% (95% CI 0.9–2.4%) [17, 18]. An unpublished mathematical model from the Institute for Disease Modeling (IDM, www.idmod.org) estimated seroprevalence to be 2% (95% CI 1%-3%) in King County for August 2020 (unpublished data; Mike Famulare, personal communication, March 23, 2021).

Seroprevalence surveys in other US jurisdictions in April and May of 2020 reported 2.5% (Georgia) [7], 2.8% (Santa Clara County, CA) [19], and 4.7% (Los Angeles County, CA) [20]. In Indiana, seroprevalence was 1.09% in a mostly White random sample and 5.8% in a mostly Black and other People of Color non-random sample [21]. It is difficult to compare results across surveys because of different sampling and analysis strategies and survey timing. We conducted our study three to four months later than these others, at a time when the county and country were in the midst of a then-unprecedented surge in cases. For both these reasons, it is expected that more cases would have accumulated and seroprevalence would be higher than in April or May. At the same time, there is evidence to suggest that antibody levels may fall below detectable limits after three to four months [22], so participants in our survey who were infected in the early months of the epidemic may no longer have had detectable levels of antibody. It is important to note that antibody status alone does not predict immunity to COVID-19; strong evidence exists that T-cell memory persists over the long term [23]. People with documented antibody should still get vaccinated and continue to follow current public health guidance regarding the wearing of masks, room ventilation, and maintaining an appropriate distance in public.

As noted above, nine participants had results positive only for the first of the two sequential assays. These may represent either false positives on the first assay or, what is more likely based on the sensitivity of the assays, false negatives on the second test. However, if the test results are accurate, these may have been individuals who were infected with SARS-CoV-2 too recently prior to the test to have developed a detectable IgG response, or who generated a response to the nucleocapsid but not the spike protein, or whose response was atypical (either weak overall or deficient in the IgG isotype [24]).

The estimated underreporting multiplier for infection based on this survey– 5—is lower than the estimates of 8.3 to 33.2 based on community serosurveys from April and May 2020 in Los Angeles, California, Miami-Dade County, Florida, Dekalb-Fulton counties, Georgia, and the states of Indiana and New York [3], and also lower than a published estimate of 7 infections per confirmed case in Washington State as of April 20, 2020 [4]. These discrepancies may reflect in part the timing of our survey in August; the pace of viral testing increased across the country in summer 2020, which would tend to close the gap between reported cases and actual infections.

In fact, our estimate of an underreporting multiplier of 5 aligns well with a mathematical model from IDM, which estimated on average 4.3 infections for every reported case in King County through mid-August [25]. Of note, the IDM model estimated that in the early part of the outbreak, from mid-March to June, there were about 8.3 infections for every case, but that by mid-July, this ratio had dropped to about 3.4 –coinciding “with the June 5 roll-out of free, drive-thru testing sites in King County, suggesting that changes in testing strategy significantly improved the infection-detection system” [25].

Goals for this survey were ambitious: we hoped to quickly generate a seroprevalence estimate for King County that would include large enough numbers of people to be able to estimate seroprevalence in all age, income, race, and ethnicity groups as well as information about social risk factors for COVID-19 that might be modifiable through behavior or policy changes.Unfortunately, we encountered serious limitations that affected timeliness and quality of the data, and we did not get useful information about modifiable social risk factors.

In addition to these and other implementation challenges, we faced more general limitations, such as differential participation by class, race, sex, and age–participants were disproportionately wealthy, White, female, and older compared to the King County population. In both the random and non-random samples, there was participation bias that worked in both directions: we were aware both of people who were ill at the time they were scheduled to be tested who did not attend and provide a blood specimen as well as people who had had an illness they suspected was COVID-19 and made a special effort to get tested.

Limitations included:

- Resources: this effort was under-resourced, with only one regular full-time PHSKC employee and one full-time consultant leading the activity. The survey was accomplished due to the huge efforts of at least 138 clinical and non-clinical Public Health Reserve Corps (PHRC) volunteers.
- Questionnaire: the questionnaire was long and the framing for some of the questions was inadequate, both of which may have discouraged participants from completing it. The sequencing of some potentially intrusive questions (e.g., about household income) was poor, some skip patterns and required questions were incorrectly implemented and/or inconsistent across the different versions of the questionnaire. Because this was a federally funded project, we were required to use federal language on race and ethnicity as well as on sexual orientation and gender identity. However, we know that these categories are not sufficiently nuanced to describe participants’ identities [26]; in particular, participants who identified as Hispanic ethnicity frequently chose “Prefer not to say” for race.
- Quality Control: About 10% of dried blood spot specimens did not have sufficient quantity of blood to be tested by the lab.
- Assay performance: The use of dried blood spots rather than serum may have caused some decline in assay performance.
- Community outreach and engagement: We did not attain our goal of oversampling members of the American Indian [6] and Pacific Islander populations despite efforts to reach out to and engage with them in conjunction with leaders from within those communities.
- Finally, the reported between-group comparisons contain additional uncertainty due to imputed demographic characteristics that were treated as known. However, further analyses (not shown here) indicate that directionality and significance of the prevalence comparisons would not be affected by re-imputation.

Despite these limitations, we believe these data provide important point estimates of SARS CoV-2 seroprevalence in the community, which we were able to disaggregate by race, income, age, and sex assigned at birth. Similar to reported case counts, seroprevalence estimates are notably higher among those with lower incomes, people of color, and–something not widely demonstrated at the time of this survey—young adults. Unlike case counts, our findings are less likely to be biased by differential testing rates and allow for more accurate estimates of the proportion of severe cases and of the infection fatality ratio.

Of course, many more cases have occurred in King County since the time of this survey; in December 2020, the average number of reported daily cases rose to 7 times the level of August 2020 [2]. As of mid-January 2021, the National Commercial Laboratory Seroprevalence Study estimate for Washington State had risen to 9.1% (95% CI 7.0%-11.2%) [16]. Seattle, which accounts for one third of King County’s population, so far has one of the lowest incidence rates of COVID-19 of any major metropolitan area in the country [27]. The relatively low underreporting factor revealed by this survey suggests that King County is testing and detecting a relatively high proportion of COVID-19 cases.

Results from this survey, combined with those of surveillance systems and other researchers, provide documentation of the extent and differential spread of SARS-CoV-2 in King County. Additional analyses may provide further insights to support messaging and policies to reduce risks.

Study data were collected and managed using REDCap electronic data capture tools hosted at the University of Washington by the Institute of Translational Health Science (ITHS) with grant support **(UL1 TR002319, KL2 TR002317, and TL1 TR002318 from NCATS/NIH)** [28, 29]. REDCap (Research Electronic Data Capture) is a secure, web-based software platform designed to support data capture for research studies, providing 1) an intuitive interface for validated data capture; 2) audit trails for tracking data manipulation and export procedures; 3) automated export procedures for seamless data downloads to common statistical packages; and 4) procedures for data integration and interoperability with external sources.

## Supporting information

S1 File English P1 questionnaire. This PDF shows the full version of the English-language questionnaire for the first person in a household. Note that this static format doesn’t capture skip patterns–most respondents would answer only a subset of questions. Likewise, the PDF does not indicate which questions were required. After survey completion we discovered inconsistencies across the 22 versions (11 English and 11 Spanish, one each for the first person in the household and adolescent/adult and child versions of the five other surveys) that affected the data quality.
(PDF)

S2 File Prevalence estimation report. (HTML)

S3 File Cleaned survey data. (CSV)

S4 File Codebook. (CSV)

S5 File Income sex distribution. Frequency of income categories by sex.
(CSV)

S6 File Age sex distribution. Frequency of age categories by sex.
(CSV)

S7 File Race sex distribution. Frequency of race categories by sex.
(CSV)

S8 File Ethnicity sex distribution. Frequency of Hispanic ethnicity by sex.
(CSV)

S9 File Income household distribution. Frequency of income categories by number in household.
(CSV)
