# Supplementary material for: Anti-SARS-CoV-2 seroprevalence in King County, WA—Cross-sectional survey, August 2020
Source: PLoS One. 2022 Aug 22;17(8):e0272783. doi: 10.1371/journal.pone.0272783 (PMC9394797; doi:10.1371/journal.pone.0272783)
Supplement: S1 File — This PDF shows the full version of the English-language questionnaire for the first person in a household. Note that this static format doesn’t capture skip patterns–most respondents would answer only a subset of questions. Likewise, the PDF does not indicate which questions were required. After survey completion we discovered inconsistencies across the 22 versions (11 English and 11 Spanish, one each for the first person in the household and adolescent/adult and child versions of the five other surveys) that affected the data quality. (PDF) [file pone.0272783.s001.pdf]

# COVID-19 Antibody Testing Study (P1)

Please complete the survey below.

Thank you!

## About your household

Thank you for participating in this survey! The survey questions ask about many factors that may affect your risk of illness with COVID-19. Some of these are related to your work or health. We are collecting this information only for the purpose of this survey. Your data will only be available to a very few persons who are conducting this project. We will protect the privacy of your data and not share your contact information or any other information for any purposes except this COVID-19 project. You may choose not to answer a question and you may stop at any time. You may also come back to the survey if you wish to finish it later.
The following set of questions is meant to be completed by one adult member of your household, and includes questions about the entire household. Afterwards, there are individual surveys for each household member. The first individual survey, also to be done by an adult, will pop up after finishing this one. If there are children in your household, their surveys will appear next. The final part is to schedule specimen collection (a few drops of blood). If, due to physical or mental issues, any adult cannot complete the individual survey themselves, another adult may complete it for them.

Name of person completing this survey: ______________________

Street address of household: ______________________
(street address (including apt if applicable))

City or town: ______________________

Zip code: ______________________

Which best describes your primary residence:
- ◯ A one-family house
- ◯ A two-family house/duplex
- ◯ An apartment building
- ◯ A dormitory
- ◯ A mobile or manufactured home
- ◯ Unhoused or homeless
- ◯ Other

Please specify what type of dwelling? ______________________

How many total apartments are there in your building? ______________________
(Please estimate if unsure.)

How many rooms are there in your primary residence? (exclude: bathrooms, laundry rooms, hallways, unfinished basements, and porches) ______________________
(Please estimate if unsure.)

How many of these rooms are bedrooms? ______________________

| | |
|---|---|
| How many people live in your household (including you)? | ◯ 1<br>◯ 2<br>◯ 3<br>◯ 4<br>◯ 5<br>◯ 6<br>◯ 7 or more |

Please answer the following questions for up to 6 members of your household. If there are 7 or more household members please answer for the oldest 6 people. For most questions, if you choose not to answer, you may skip to the next question. A small number of questions are required to participate in this project.

We are collecting this information only for the purpose of this survey. Your data will only be available to a very few persons who are conducting this project. We will protect the privacy of your data and not share your contact information, or any other information for any other purposes besides this COVID project.

For children under the age of 13, we will not collect contact information; a parent or guardian must complete for each child 12 years of age and under. If a teenager or adult cannot complete the survey due to a physical or mental condition, any adult member of the household may complete the survey for them, and please enter that adult's (the one completing the survey) contact information.

| | |
|---|---|
| How old are you? | ________________________ |
| What are your initials? | ________________________ |
| If you have a cell phone, what is that phone number? | ________________________ |
| If you have a landline phone, what is that phone number? | ________________________ |
| What is your email address? This is the email address that we will send your COVID antibody test results to unless you request a different method; we will also use this email address for returning the antibody results for any children (age 12 and under) in your household. | ________________________ |
| How would you like to receive your test results? | ◯ Email to [email1]<br>◯ Text to [cell1]<br>◯<br>◯ Call to [landline1]<br>◯ Letter to [address] |
| What are person 2's initials? | ________________________ |
| What is [initials2]'s age? | ________________________ |
| How many months old is [initials2]? | ________________________<br>(Please enter how many months old if this child is 2 years old or younger) |

| | |
|---|---|
| What is your relationship to [initials2]? | ◯ Partner or spouse<br>◯ Child (including biological, adopted, stepchild, or foster child)<br>◯ Sibling (biological or other)<br>◯ Parent (biological or other)<br>◯ Other relative (for example aunt, uncle, grandparent or grandchild)<br>◯ Roommate or housemate<br>◯ Other non-relative (for example, a live in caretaker) |
| If [initials2] has a cell phone, please enter that number | ________________________________ |
| Please enter [initials2]'s email address | ________________________________ |
| What are person 3's initials? | ________________________________ |
| What is [initials3]'s age? | ________________________________ |
| How many months old is [initials3]? | ________________________________<br>(Please enter how many months old if this child is 2 years old or younger) |
| What is your relationship to [initials3]? | ◯ Partner or spouse<br>◯ Child (including biological, adopted, stepchild, or foster child)<br>◯ Sibling (biological or other)<br>◯ Parent (biological or other)<br>◯ Other relative (for example aunt, uncle, grandparent or grandchild)<br>◯ Roommate or housemate<br>◯ Other no-relative (for example, a live in caretaker) |
| If [initials3] has a cell phone, please enter that number | ________________________________ |
| Please enter [initials3]'s email address | ________________________________ |
| What are person 4's initials? | ________________________________ |
| What is [initials4]'s age? | ________________________________ |
| How many months old is [initials4]? | ________________________________<br>(Please enter how many months old if this child is 2 years old or younger) |

| | |
|---|---|
| What is your relationship to [initials4]? | ◯ Partner or spouse<br>◯ Child (including biological, adopted, stepchild, or foster child)<br>◯ Sibling (biological or other)<br>◯ Parent (biological or other)<br>◯ Other relative (for example aunt, uncle, grandparent or grandchild)<br>◯ Roommate or housemate<br>◯ Other no-relative (for example, a live in caretaker) |
| If [initials4] has a cell phone, please enter that number | __________________________ |
| Please enter [initials4]'s email address | __________________________ |
| What are person 5's initials? | __________________________ |
| What is [initials5]'s age? | __________________________ |
| How many months old is [initials5]? | __________________________<br>(Please enter how many months old if this child is 2 years old or younger) |
| What is your relationship to [initials5]? | ◯ Partner or spouse<br>◯ Child (including biological, adopted, stepchild, or foster child)<br>◯ Sibling (biological or other)<br>◯ Parent (biological or other)<br>◯ Other relative (for example aunt, uncle, grandparent or grandchild)<br>◯ Roommate or housemate<br>◯ Other no-relative (for example, a live in caretaker) |
| If [initials5] has a cell phone, please enter that number | __________________________ |
| Please enter [initials5]'s email address | __________________________ |
| What are person 6's initials? | __________________________ |
| What is [initials6]'s age? | __________________________ |
| How many months old is [initials6]? | __________________________<br>(Please enter how many months old if this child is 2 years old or younger) |

| | |
|---|---|
| What is your relationship to [initials6]? | ◯ Partner or spouse<br>◯ Child (including biological, adopted, stepchild, or foster child)<br>◯ Sibling (biological or other)<br>◯ Parent (biological or other)<br>◯ Other relative (for example aunt, uncle, grandparent or grandchild)<br>◯ Roommate or housemate<br>◯ Other no-relative (for example, a live in caretaker) |
| If [initials6] has a cell phone, please enter that number | __________________________________ |
| Please enter [initials6]'s email address | __________________________________ |

If any household members are age 13 years and older and you have provided an email address for them, a link to the individual survey will be sent to them. If you have not provided an email address for any teens or adults, but have provided a cell phone number, a link may be sent by text to that cell phone. And if you have provided neither, a link will be sent to you.

| | |
|---|---|
| Since March 1, 2020, how many guests has your household typically received per week? A guest is anyone who comes into your home but does not live in your household (could include a repair technician, social worker, or other professional as well as non-household family members or friends). | ◯ None<br>◯ Less than 1 per week<br>◯ 1-2 per week<br>◯ 3-4 per week<br>◯ 5 or more per week |
| What was your total household income in 2019 from all sources (please estimate if unsure)? | ◯ < $20,000<br>◯ $20,000 - $40,000<br>◯ $40,001 - $60,000<br>◯ $60,001 - $80,000<br>◯ $80,001 - $100,000<br>◯ $100,001 - $120,000<br>◯ $120,001+ |
| Including you, how many people depend on this income? | ◯ 1<br>◯ 2<br>◯ 3<br>◯ 4<br>◯ 5<br>◯ 6<br>◯ 7 or more |
| Please check all sources of your current household income | ☐ Wages/salary<br>☐ Tips/bonuses<br>☐ Unemployment benefits<br>☐ Social security<br>☐ Other government income assistance (GAU, TANF, SNAP, SSI, Disability, TANF, WIC)<br>☐ Income from assets (stocks/bonds, rental homes, annuities, etc)<br>☐ Other (pension, annuity, savings, gifts, etc.) |
| Have any members of your household died since January 1, 2020? | ◯ Yes<br>◯ No |

| Question | Response |
|---|---|
| We send our condolences for your loss. Did your household member who died have COVID-19 symptoms or a COVID-19 diagnosis? | ◯ Yes<br>◯ No |

Thank you for completing this part. Next there is an individual survey. It has questions about you and any COVID-like illness you may have had and your risks for COVID-19. After that, you can schedule a time that we will collect your sample for antibody testing (a few drops of blood).

Thank you for completing this short survey about your household. Next there is a survey about covid for each household member, starting with you! The survey includes questions about any COVID-like illnesses you may have had and your risks for COVID-19. After that, you can schedule a time that we will collect your (or your household members') sample for antibody testing (a few drops of blood).

Thank you for participating in this survey! The survey questions ask about many factors that may affect your risk of illness with COVID-19. Some of these are related to your work or health. We are collecting this information only for the purpose of this survey. Your data will only be available to a very few persons who are conducting this project. We will protect the privacy of your data and not share your contact information or any other information for any purposes except this COVID-19 project. You may choose not to answer a question and you may stop at any time. You may also come back to the survey if you wish to finish it later.
This questionnaire is meant to be completed by each adult member of the household. If, due to physical or mental issues any member is unable to complete this form themselves, another adult may do it for them.

| Question | Response |
|---|---|
| What are your initials (or the person you are answering for)? | __________________________________ |
| How old are you (or the person you are answering for)? | __________________________________ |
| What sex were you assigned at birth, or on your original birth certificate? | ◯ Male<br>◯ Female<br>◯ I prefer not to answer<br>◯ I don't know |
| Do you currently describe yourself as male, female, or transgender? | ◯ Male<br>◯ Female<br>◯ Transgender<br>◯ None of these |
| How concerned are you about being exposed to COVID-19 in your home? | ◯ Not at all concerned<br>◯ Slightly concerned<br>◯ Moderately concerned<br>◯ Very concerned<br>◯ Extremely concerned<br>◯ Not applicable |
| What was your typical kind of transportation before March 1, 2020? (check all that apply) | ☐ Drive in a car or truck alone<br>☐ In a car or truck with one or more other person<br>☐ Public bus<br>☐ Train or light rail<br>☐ Uber, Lyft, or Taxi<br>☐ Bike or walk<br>☐ Ferry<br>☐ Other |

| | |
|---|---|
| How concerned would you be about your exposure to COVID-19 if you used your typical kinds of transportation today? | ◯ Not at all concerned<br>◯ Slightly concerned<br>◯ Moderately concerned<br>◯ Very concerned<br>◯ Extremely concerned<br>◯ Not applicable |
| Have you worked outside the home or been a student in a classroom SINCE MARCH 1, 2020? | ◯ Yes<br>◯ No |
| How concerned are you about your exposure to COVID-19 at your workplace or school SINCE MARCH 1, 2020? | ◯ Not at all concerned<br>◯ Slightly concerned<br>◯ Moderately concerned<br>◯ Very concerned<br>◯ Extremely concerned<br>◯ Not applicable |

## Information about recent illness

| | |
|---|---|
| Do you think you have had a COVID-19 infection? | ◯ Yes<br>◯ No |
| How much did you stay at home because of your illness? Please think of your most recent episode if you have had more than one COVID-like illness. | ◯ None of the time<br>◯ Some of the time<br>◯ All of the time |
| During your illness, did you stay in a bedroom by yourself? | ◯ Yes<br>◯ No |
| During your illness, did you have a bathroom that only you used? | ◯ Yes<br>◯ No |
| When was the first day that you began to feel sick (please estimate if unsure)? | __________________________ |
| Do you still feel sick? | ◯ Yes<br>◯ No |
| When was the first day that you no longer felt sick (please estimate if unsure)? | __________________________ |
| During the time that you were sick, which of the following symptoms did you have? | |
| Fever as measured by a thermometer | ◯ No fever<br>◯ Mild (99.5-100.9F or 37.5-38.3C )<br>◯ Moderate to high (101.0-103.9F or 38.4-39.9C)<br>◯ Very high (104.0F or 40.0C or more) |
| Felt feverish | ◯ Did NOT feel feverish<br>◯ Mild<br>◯ Moderate<br>◯ Severe |
| Chills | ◯ No chills<br>◯ Mild<br>◯ Moderate<br>◯ Severe |

| | |
|---|---|
| Cough | ◯ No cough<br>◯ Mild<br>◯ Moderate<br>◯ Severe |
| Sore throat | ◯ No sore throat<br>◯ Mild<br>◯ Moderate<br>◯ Severe |
| Runny or stuffy nose | ◯ No runny/stuffy nose<br>◯ Mild<br>◯ Moderate<br>◯ Severe |
| Difficulty breathing | ◯ No difficulty breathing<br>◯ Mild<br>◯ Moderate<br>◯ Severe |
| Muscle pain | ◯ No muscle pain<br>◯ Mild<br>◯ Moderate<br>◯ Severe |
| Chest pain | ◯ No chest pain<br>◯ Mild<br>◯ Moderate<br>◯ Severe |
| Stomach pain | ◯ No stomach pain<br>◯ Mild<br>◯ Moderate<br>◯ Severe |
| Nausea or vomiting | ◯ No nausea or vomiting<br>◯ Mild<br>◯ Moderate<br>◯ Severe |
| Diarrhea | ◯ No diarrhea<br>◯ Mild<br>◯ Moderate<br>◯ Severe |
| Headache | ◯ No headache<br>◯ Mild<br>◯ Moderate<br>◯ Severe |
| Fatigue or felt very tired | ◯ No fatigue<br>◯ Mild<br>◯ Moderate<br>◯ Severe |

| | |
|---|---|
| Loss of smell or taste | ◯ No loss of taste or smell<br>◯ Mild<br>◯ Moderate<br>◯ Severe |
| Any other symptoms? | ◯ No symptoms other than those above<br>◯ Mild<br>◯ Moderate<br>◯ Severe |
| What was (or were) the other symptom(s)? | ______________________ |
| Did you go to a doctor, clinic, or emergency room because of this illness? | ◯ Yes<br>◯ No<br>◯ Unsure |
| Did you stay overnight in the hospital for this illness? | ◯ Yes<br>◯ No<br>◯ Unsure |
| How many days were you hospitalized ? | ______________________<br>(Days) |
| How many days after you noticed your symptoms were you admitted to the hospital? | ______________________<br>(Days) |
| Were you tested for COVID-19 during this illness? | ◯ No<br>◯ Yes<br>◯ Unsure |
| On approximately which date were you tested? (Please estimate if unsure) | ______________________<br>(MM/DD/YYYY) |
| What was your test result? | ◯ Negative<br>◯ Positive<br>◯ Unknown or inconclusive |
| Did you receive a diagnosis for this illness? | ◯ No<br>◯ Yes<br>◯ Unsure |
| What was the diagnosis? | ______________________ |
| Why did you NOT get a test for COVID-19 during your illness? | ☐ Mild illness<br>☐ My medical provider did not offer or recommend a test<br>☐ Testing was not available to me<br>☐ I did not want to give the virus others<br>☐ I did not want to be get the virus from others<br>☐ I could not afford it or insurance did not cover it<br>☐ I did not want to know<br>☐ Other |

| | |
|---|---|
| Please specify what other reasons? | __________________________________ |
| Did you miss any days of work (or school) because of this illness? | ◯ No<br>◯ Yes<br>◯ Unsure or don't remember |
| How many days of work (or school) did you miss? | __________________________________ |

## Medical history

Do you have any of the following medical conditions?

| | |
|---|---|
| Seasonal allergies or hay fever | ◯ Yes<br>◯ No |
| Chronic lung disease | ◯ Yes<br>◯ No |
| Asthma or reactive airway disease | ◯ Yes<br>◯ No |
| Emphysema or COPD (Chronic obstructive pulmonary disease) | ◯ Yes<br>◯ No |
| Other lung disease (for example, active tuberculosis/TB) | ◯ Yes<br>◯ No |
| Please specify what other lung disease ? | __________________________________ |
| Diabetes | ◯ Yes<br>◯ No |
| Well controlled diabetes (HbA1c consistenly < 7.0) | ◯ Yes<br>◯ No |
| Heart or cardiovascular disease | ◯ Yes<br>◯ No |
| High blood pressure | ◯ Yes<br>◯ No |
| Well controlled high blood pressure (consistently < 130/< 80) | ◯ Yes<br>◯ No |
| Coronary artery disease | ◯ Yes<br>◯ No |
| Heat failure or congestive heart disease | ◯ Yes<br>◯ No |
| Stroke | ◯ Yes<br>◯ No |

| | |
|---|---|
| Congenital heart disease | ◯ Yes<br>◯ No |
| Other disease of the heart or vascular system | ◯ Yes<br>◯ No |
| Please specify what other heart or vascular disease? | __________________________________ |
| Kidney disease | ◯ Yes<br>◯ No |
| Receiving dialysis | ◯ Yes<br>◯ No |
| Liver disease (including cirrhosis, chronic hepatitis B, or chronic hepatitis C) | ◯ Yes<br>◯ No |
| Any immune-compromising condition | ◯ Yes<br>◯ No |
| HIV | ◯ Yes<br>◯ No |
| HIV with a recent CD4 < 350 | ◯ Yes<br>◯ No |
| Solid organ transplant | ◯ Yes<br>◯ No |
| Stem cell transplant | ◯ Yes<br>◯ No |
| Cancer | ◯ Yes<br>◯ No |
| Taking drugs that suppress the immune system (like steroids) | ◯ Yes<br>◯ No |
| Neurologic/neurodevelopmental disorder (for example, a traumatic brain injury) | ◯ Yes<br>◯ No |
| Mental or behavioral health condition (including severe depression, bipolar disorder, schizophrenia, or addictions) | ◯ Yes<br>◯ No |
| Sickle cell disease (not including carrier status) | ◯ Yes<br>◯ No |
| Thalessemia (not including carrier status) | ◯ Yes<br>◯ No |
| Cystic fibrosis (not including carrier status) | ◯ Yes<br>◯ No |

| | |
|---|---|
| Other chronic condition | ◯ Yes<br>◯ No |
| Please specify which other chronic condition(s)? | ______________________________ |
| My current smoking status is | ◯ Never a regular smoker<br>◯ Ex-smoker<br>◯ Current smoker (including occasional smoking and e-cigarettes) |
| What is your height, starting with how many feet tall? (You can select if you prefer to use cm) | ◯ Prefer to use cm<br>◯ 2<br>◯ 3<br>◯ 4<br>◯ 5<br>◯ 6<br>◯ 7<br>(Feet) |
| And how many inches? | ◯ 1<br>◯ 2<br>◯ 3<br>◯ 4<br>◯ 5<br>◯ 6<br>◯ 7<br>◯ 8<br>◯ 9<br>◯ 10<br>◯ 11<br>(Inches) |
| What is your height in centimeters? | ______________________________<br>(Cm) |
| What do you weigh in pounds? (Please leave blank if you prefer to answer in kg). | ______________________________<br>(Pounds) |
| What is your weight in kilograms? | ______________________________ |
| Are you pregnant or have you given birth WITHIN THE PAST 6 WEEKS? | ◯ Yes<br>◯ No |
| Are you breastfeeding? | ◯ Yes<br>◯ No |
| SINCE JANUARY 1, 2020, do you think any of your household members has been ill with a COVID-19 infection? | ◯ Yes<br>◯ No |
| DURING THEIR ILLNESS, how much care did you provide to this person or people? | ◯ No care<br>◯ Some care<br>◯ Most or all care |

| | |
|---|---|
| DURING THEIR ILLNESS, how much time did you spend within 6 feet of this person or people per day? | ◯ Less than 10 minutes<br>◯ 10 to 30 minutes<br>◯ 30 to 60 minutes<br>◯ 1 to 2 hours<br>◯ More than 2 hours |
| While you were within 6 feet of the ill person or people, how often were you wearing a mask? | ◯ Never<br>◯ Rarely<br>◯ Sometimes<br>◯ Most of the time<br>◯ Always |
| Overall, are you yourself practicing social (or physical) distancing with non-household members? | ◯ Never<br>◯ Rarely<br>◯ Sometimes<br>◯ Most of the time<br>◯ Always |
| SINCE JANUARY 1, 2020 have you stayed for one or more nights in any of the following locations? Check all that apply. | ☐ Hospital<br>☐ Nursing home or assisted living facility<br>☐ Other healthcare facility (long term care facility, acute care inpatient facility, rehabilitation facility)<br>☐ Jail, prison, or detention center<br>☐ Homeless shelter<br>☐ Group home<br>☐ Hotel, motel, rental<br>☐ Friend or family member's home |
| Do you have access to a personal vehicle (such as a car, passenger class truck or van, or motorcycle)? | ◯ Yes<br>◯ No |
| IN THE PAST WEEK, how many days did you leave your home? (Please enter "0" if you have not left your home) | ____________________<br>(Number of days) |

**IN THE PAST MONTH, how often have you left your home to go to :**

| | Never | Less than once per week | About once a week | A few times a week | Every day (or nearly every day) |
|---|---|---|---|---|---|
| The grocery store | ◯ | ◯ | ◯ | ◯ | ◯ |
| A restaurant (dine in/take out) | ◯ | ◯ | ◯ | ◯ | ◯ |
| A retail store (including pharmacy or drugstore) | ◯ | ◯ | ◯ | ◯ | ◯ |
| Seek health care (including dental care, mental health, physical therapy, etc.) | ◯ | ◯ | ◯ | ◯ | ◯ |
| Walk/run/bike/other active outside activities | ◯ | ◯ | ◯ | ◯ | ◯ |
| Work | ◯ | ◯ | ◯ | ◯ | ◯ |

| | | | | | |
|---|---|---|---|---|---|
| The home of a family member, friend, or other person | ◯ | ◯ | ◯ | ◯ | ◯ |
| A social gathering | ◯ | ◯ | ◯ | ◯ | ◯ |
| Religious gathering or service | ◯ | ◯ | ◯ | ◯ | ◯ |
| A public rally/protest/march | ◯ | ◯ | ◯ | ◯ | ◯ |
| Other community gathering | ◯ | ◯ | ◯ | ◯ | ◯ |
| Some other reason to leave home | ◯ | ◯ | ◯ | ◯ | ◯ |

Please specify your other reason(s) for leaving your home?

__________________________________

IN THE PAST MONTH, how many days did you take a bus, light rail, ferry, train or any other kind of public transportation??

__________________________________

On days that you took public transportation, how many total minutes, on average, would you spend on the bus, train, and/or ferry? (Count both ways if you go round trip)

__________________________________
(Minutes)

IN THE PAST WEEK, to which of the following locations did you take public transportation? Check all that apply.

- ☐ Job/employment or school
- ☐ Grocery store, drug store, or pharmacy
- ☐ Other store
- ☐ Health care visit
- ☐ Residence of friend or family
- ☐ Religious service or community gathering
- ☐ Park or other greenspace
- ☐ Other: Please specify:

IN THE PAST WEEK, on average, how many people were in the seats immediately next to, in front of, or behind yours or standing next to you on public transportation?

- ◯ 0
- ◯ 1 to 2
- ◯ 3 to 4
- ◯ 5 or more

IN THE PAST WEEK, how often did you wear a mask while on public transportation??

- ◯ Never
- ◯ Rarely
- ◯ Sometimes
- ◯ Most of the time
- ◯ Always

What best describes your employment status SINCE MARCH 1, 2020?

- ◯ Employed, fully remote
- ◯ Employed, fully on-site
- ◯ Employed, partly remote, partly on-site
- ◯ Unemployed or fully retired

| | |
|---|---|
| Do you work in any of the following locations | ☐ Hospital<br>☐ Nursing home or assisted living facility<br>☐ Other healthcare facility (long term care facility, acute care inpatient facility, rehabilitation facility)<br>☐ Jail, prison, or detention center<br>☐ Homeless shelter<br>☐ Group home<br>☐ Grocery store<br>☐ Fire station or ambulance<br>☐ Mail, package, or meal delivery<br>☐ Bus driver, ride share, or public transportation |
| Do you work in any of the following sectors | ☐ Home health<br>☐ Other medical, dental, or counseling<br>☐ Public transportation<br>☐ Retail and customer service<br>☐ Food service and delivery<br>☐ Public safety and emergency response<br>☐ Agriculture and food supply<br>☐ Packing and shipping<br>☐ Day care<br>☐ School<br>☐ Other high-risk location or sector |
| Please specify what other sector you work in? | ______________________________ |
| SINCE MARCH 1, 2020 how many hours do you typically work in a week? | ______________________________<br>(Hours) |
| SINCE MARCH 1, 2020 what best describes your work schedule? | ◯ Traditional or days (start between 6 am  - noon)<br>◯ Swing shift or evenings/nights (start between 1 pm - 8 PM)<br>◯ Graveyard or nights/early mornings (start between 9 PM-5 AM)<br>◯ Variable (no set schedule, work at different times -- early mornings, days, and nights) |
| How is your wage paid? | ☐ Salaried<br>☐ Hourly<br>☐ Commissions/Sales<br>☐ Per client<br>(Check all that apply) |
| Are you part of a union? | ◯ Yes<br>◯ No |
| How have your hours worked changed SINCE MARCH 1, 2020? SINCE MARCH 1, 2020 I now work: | ◯ Fewer hours<br>◯ The same hours, no change<br>◯ More hours |
| What percent of the time do you currently work remotely? | ◯ Not at all, 0%<br>◯ Less than 25%<br>◯ 25%-49%<br>◯ 50%-74%,<br>◯ 75% or more but less than 100%<br>◯ 100% |

| | |
|---|---|
| IN THE PAST WEEK, how many minutes did it typically take you to get from home to work (each way)? | ______________________ (Minutes) |
| Does your work involve direct contact with known or suspected COVID-19 patients? | ◯ Yes<br>◯ No |
| What types of personal protective equipment (PPE) were available to you at work IN THE PAST WEEK? | ☐ Mask<br>☐ Face shield or goggles<br>☐ Gloves<br>☐ Hand sanitizer<br>☐ Physical barrier or divider<br>☐ Gown<br>☐ None<br>☐ Other: Please specify:<br>(Check all that apply) |
| Please specify | ______________________ |
| IN THE PAST WEEK, did you use the personal protective equipment (PPE) that was available to you? | ◯ Never<br>◯ Sometimes<br>◯ Always<br>◯ No PPE available |
| IN THE PAST WEEK, how often did you wash your hands or use hand sanitizer at work? | ◯ Less than 1 time per day<br>◯ 1-3 times per day<br>◯ 4-9 times per day<br>◯ 10 or more times per day |
| What other precautions has your employer taken SINCE MARCH 1, 2020 to address COVID-19? Check all that apply. | ☐ Increased sanitation and cleaning of workplace<br>☐ Work in shifts<br>☐ Social distancing markers<br>☐ Increased space between employees<br>☐ Temperature or symptom screening<br>☐ Enabled some workers to work remotely<br>☐ None<br>☐ Other<br>(Check all that apply) |
| Please specify what other measures your employer has instituted? | ______________________ |
| IN THE PAST WEEK, have you been in close contact (defined as face-to-face within 6 feet for 15 minutes or more) with the public as part of your job? | ◯ Yes<br>◯ No |
| How many customers (or members of the public) came within 6 feet of you at your job in an average hour IN THE PAST WEEK? | ◯ Fewer than 1 per hour<br>◯ 1-3 per hour<br>◯ 4-9 per hour<br>◯ 10 or more per hour |
| How many of these customers were wearing masks? | ◯ None<br>◯ Less than half<br>◯ More than half<br>◯ All |

| | |
|---|---|
| IN THE PAST WEEK, were you in close contact (defined as face-to-face within 6 feet for 15 minutes or more) with your co-workers as part of your job? | ◯ Yes<br>◯ No |
| How many co-workers came within 6 feet of you at your job in an average hour IN THE PAST WEEK? | ◯ Fewer than 1 per hour<br>◯ 1-3 per hour<br>◯ 4-9 per hour<br>◯ 10 or more per hour |
| How many of these co-workers were wearing masks? | ◯ None<br>◯ Less than half<br>◯ More than half<br>◯ All |
| SINCE MARCH 1, 2020, how many definite COVID-19 cases are you aware of at your workplace? | ________________________ |
| How many other COVID-19 cases do you believe there have been at your workplace? (not counting definite cases) | ________________________ |
| How concerned are you about your co-workers coming into work while sick with COVID-19?? | ◯ Not at all concerned<br>◯ Slightly concerned<br>◯ Moderately concerned<br>◯ Very concerned<br>◯ Extremely concerned<br>◯ Not applicable |
| Which of these policies does your employer offer? | ☐ Paid sick leave<br>☐ Unpaid sick leave<br>☐ Paid work-from-home<br>☐ Paid overtime<br>☐ Hazard pay<br>☐ None<br>(Check all that apply.) |
| I would be penalized by my employer for staying home sick from work. | ◯ Yes<br>◯ No |
| I cannot afford to stay home sick from work. | ◯ Yes<br>◯ No |
| Have you previously been tested for COVID-19? (Or if you got a negative test with an illness, have you had an additional test?) | ◯ Yes<br>◯ No |
| What was the result of that test? | ◯ Negative<br>◯ Positive from a nose or throat or oral swab<br>◯ Positive from a blood test (for antibodies)<br>◯ Unknown or inconclusive |
| How likely would you be able to get a COVID-19 test if you needed one? | ◯ Not at all likely<br>◯ Slightly likely<br>◯ Moderately likely<br>◯ Very likely<br>◯ Completely likely<br>◯ Unsure/don't know |

| | |
|---|---|
| Are you of Latino/Latina/Latinx/Hispanic ethnicity? | ◯ Yes<br>◯ No |
| What is your race? (Or races) | ☐ American Indian or Alaska Native<br>☐ Asian<br>☐ Black or African American<br>☐ Native Hawaiian or Pacific Islander<br>☐ White<br>☐ Prefer not to answer<br>(Please check all that apply) |
| What is/are your tribal affiliation(s)? | __________________________________ |
| Do you speak a language other than English at home? | ◯ Yes<br>◯ No |
| What is the main language you speak at home (other than English)? | __________________________________ |
| Do you currently have health insurance? | ☐ Yes, private or through my work/family member's work<br>☐ Yes, public (Apple Health, Medicaid, Medicare, etc)<br>☐ No<br>(Check all that apply) |
| What is the highest level of education that you have completed? | ◯ 8th grade or less<br>◯ Some high school, but did not graduate<br>◯ High school graduate or GED<br>◯ Technical or vocational school<br>◯ Some college or 2-year degree<br>◯ 4-year college degree<br>◯ More than 4-year college degree |
| Which of the following best represents how you think of yourself? | ◯ Lesbian or gay<br>◯ Straight, that is not lesbian or gay<br>◯ Bisexual<br>◯ Something else<br>◯ Prefer not to answer<br>◯ I don't know the answer |
| Please specify your sexual orientation? | __________________________________ |
| Were you born in the US? | ◯ Yes<br>◯ No |
| If no, what country were you born in? | __________________________________ |

Thank you for your time and participation today. Your responses will be used to identify risk factors for COVID-19 exposure and to design public health interventions to make our homes, transportation systems, and workplaces safer for all of us. Please fill out the survey for each child in the household and we will send the survey on to each adult or teenager.

Please answer the next few questions to set up a time to donate your specimen (a few drops of blood) for antibody testing. Below are the dates, places, and addresses for drive-through testing.

1. Sat 8/8 Federal Way: 31405 18th Ave S, Federal Way, WA 98003 (Sea Mar)
https://goo.gl/maps/89C6CraF67TQHy2s6

2. Sun 8/9 Aurora: 12040 Aurora Ave N, Seattle 98133 (old emissions testing site)
https://goo.gl/maps/EvM4ei1FScj4CaRP7

3. Sun 8/9 SODO: 3820 6th Ave S., Seattle, WA 98108 (old emissions testing site)
https://goo.gl/maps/EXsfkcByrvUU9Mup9

4. Sat 8/15 Eastgate: 14350 SE Eastgate Way, Bellevue, 98007 (Public Health Clinic)
https://goo.gl/maps/4X2g8df79nMTR49r9

5. Sun 8/16 Aurora: 12040 Aurora Ave N, Seattle 98133 (old emissions testing site)
https://goo.gl/maps/EvM4ei1FScj4CaRP7

| | |
|---|---|
| Do you have a car or a way to get to one of the testing sites (as above) ? | ◯ Yes<br>◯ No |
| Which time and place is best for your household to get a specimen collected? (This should be a time and place that all members of your household willing to participate can attend.) | ◯ Saturday August 8, 10am - 2pm Federal Way<br>◯ Saturday August 8, 2pm - 6pm Federal Way<br>◯ Sunday August 9, 10 am - 2 pm Aurora<br>◯ Sunday August 9, 10 am - 2 pm SODO<br>◯ Sunday August 9, 2 pm - 6 pm Aurora<br>◯ Sunday August 9, 2 pm - 6 pm SODO<br>◯ Saturday August 15, 10am - 2 pm Eastgate<br>◯ Saturday August 15, 2 pm - 6 pm Eastgate<br>◯ Sunday August 16, 10 am - 2 pm Aurora<br>◯ Sunday August 16, 2 pm - 6 pm Aurora |
| Which specific time is best for you? | ◯ 10:00 am<br>◯ 10:15 am<br>◯ 10:30 am<br>◯ 10:45 am<br>◯ 11:00 am<br>◯ 11:15 am<br>◯ 11:30 am<br>◯ 11:45 am<br>◯ 12:00 noon<br>◯ 12:15 pm<br>◯ 12:30 pm<br>◯ 12:45 pm<br>◯ 1:00 pm<br>◯ 1:15 pm<br>◯ 1:30 pm<br>◯ 1:45 pm |

| | |
|---|---|
| Which specific time is best for you? | ◯ 10:00 am<br>◯ 10:15 am<br>◯ 10:30 am<br>◯ 10:45 am<br>◯ 11:00 am<br>◯ 11:15 am<br>◯ 11:30 am<br>◯ 11:45 am<br>◯ 12:00 noon<br>◯ 12:15 pm<br>◯ 12:30 pm<br>◯ 12:45 pm<br>◯ 1:00 pm<br>◯ 1:15 pm<br>◯ 1:30 pm<br>◯ 1:45 pm |
| Which specific time is best for you? | ◯ 2:00 pm<br>◯ 2:15 pm<br>◯ 2:30 pm<br>◯ 2:45 pm<br>◯ 3:00 pm<br>◯ 3:15 pm<br>◯ 3:30 pm<br>◯ 3:45 pm<br>◯ 4:00 pm<br>◯ 4:15 pm<br>◯ 4:30 pm<br>◯ 4:45 pm<br>◯ 5:00 pm |
| Which specific time is best for you? | ◯ 2:00 pm<br>◯ 2:15 pm<br>◯ 2:30 pm<br>◯ 2:45 pm<br>◯ 3:00 pm<br>◯ 3:15 pm<br>◯ 3:30 pm<br>◯ 3:45 pm<br>◯ 4:00 pm<br>◯ 4:15 pm<br>◯ 4:30 pm<br>◯ 4:45 pm<br>◯ 5:00 pm |
| What date and time interval would be best for you to have our testers come to your household? This should be a date that all household members willing to participate can be present. We will do our best to contact you beforehand to give you a better estimate of when to expect us. Due to the size of King County, we may not be able to estimate very accurately when we can be at your home. When we get there, we will collect your specimen outdoors: in a front yard, back yard, an apartment building courtyard, or other nearby site. | ◯ Monday Aug 10, 11 am-3 pm (Home visit)<br>◯ Monday Aug 10, 3-7 pm (Home visit)<br>◯ Tuesday Aug 11, 11 am-3 pm (Home visit)<br>◯ Tuesday Aug 11, 3-7 pm (Home visit)<br>◯ Wednesday Aug 12, 11 am-3 pm (Home visit)<br>◯ Wednesday, Aug 12, 3-7 pm (Home visit)<br>◯ Thursday Aug 13, 11 am-3 pm (Home visit)<br>◯ Thursday, Aug 13, 3-7 pm (Home visit)<br>◯ Friday Aug 14, 11 am-3 pm (Home visit)<br>◯ Friday Aug 14, 3-7 pm (Home visit) |
| Are there any special instructions our team should know before we come to your home? (For example, don't let the dog out of the yard, or come to the door on the left side of the house.) | ________________________________ |

We will send a confirmation with the time and place for your test.
If, due to work schedules, or vehicle or household size, it is not possible for all household members who would like to participate to come to a screening site together, please have the remainder of your household come to any of the collection sites as a "walk in" or unscheduled participant.
Thank you!

Name:

Please enter your name and date of birth if you wish to get an antibody test. Your name and date of birth will serve as your consent to allow antibody testing. Your information will be kept confidential and is required by the laboratory (this is standard laboratory practice).

______________________________

Date of birth

______________________________

If [initials2] might also consent to have an antibody test please list their full name.

______________________________

[initials2]'s date of birth

______________________________

If [initials3] might also consent to have an antibody test please list their full name.

______________________________

[initials3]'s date of birth

______________________________

If [initials4] might also consent to have an antibody test please list their full name.

______________________________

[initials4]'s date of birth

______________________________

If [initials5] might also consent to have an antibody test please list their full name.

______________________________

[initials5]'s date of birth

______________________________

If [initials6] might also consent to have an antibody test please list their full name.

______________________________

[initials6]'s date of birth

______________________________
