# Supplementary material for: Anti-SARS-CoV-2 seroprevalence in King County, WA—Cross-sectional survey, August 2020
Source: PLoS One. 2022 Aug 22;17(8):e0272783. doi: 10.1371/journal.pone.0272783 (PMC9394797; doi:10.1371/journal.pone.0272783)
Supplement: S2 File — (HTML) [file pone.0272783.s002.html]

Prevalence Estimation


Code 

- Show All Code
- Hide All Code

# Prevalence Estimation

#### The Mountain Whisper Light

#### 2021-07-15

# 1 Introduction

This document outlines an estimation strategy developed to estimate the Seroprevalence of COVID19 in King County in early to mid August of 2020. The estimation makes use of the survey weights developed by Marketing Systems Group. The stratified cluster survey was carried out with two strata, a random address-based sample and a convenience sample. The survey weights have been raked to King county population characteristics (race, Hispanic status, sex, age, and income), assuming equal sampling weights for sampling units (households) within each stratum. This required imputation of missing data on the raking variables. We carry out estimation using Survey R package (Lumley 2020)

There are various methodologic challenges to account for when calculating the estimate and corresponding standard errors.

We present the final estimate based on three adjustments; one accounting for missingness, one accounting for raking, and one accounting for the sensitivity and specificity of the testing procedure.

Additional analyses were carried out to account for other possible sources of bias and variance.

**Note that all data presented here (before the appendix) have already been cleaned and processed. This includes:**

- Removal of some duplicate entries
- Construction of variables implied by survey responses

```
## Reading in data
surv_df <- read.csv("data/cleaned_survey_data.csv")
ps_obj <- list(
 age_sex  = read.csv("data/age_sex_distr.csv"),
 hisp_sex = read.csv("data/hisp_sex_distr.csv"),
 race_sex = read.csv("data/race_sex_distr.csv"),
 inc_sex  = read.csv("data/inc_sex_distr.csv"),
 inc_hhs  = read.csv("data/inc_hhs_distr.csv")
)
```

## 1.1 Missing data

The dataset contains all individuals who provided blood samples for testing for the presence of COVID19 antibody. However, some (79) individuals did not have sufficient quantities of blood to run tests. To account for this missingness, we assume the data are missing at random, and use a two phase model to approach.

## 1.2 Accounting for Raking

The weights provided for this analysis were calculated using raking. When weighted estimates are based on raking the corresponding standard errors can be reduced (see, for example chapter 7 of Lumley 2010). This is accounted for in our analysis using the R survey package.

## 1.3 Accounting for sensitivity and specificity of the two tests

The estimate (and corresponding standard error) resulting from the data and the above adjustments is for the proportion of individuals who tested positive on both tests. A prevalence estimate can be calculated from the estimate of proportion positive if the sensitivity and specificity of the combined test are known using the following formula:

\[
\text{Prevalence} = \frac{\text{Proportion Positive} + \text{Specificity} - 1 }{\text{Sensitivity} + \text{Specificity} - 1}
\]

## 1.4 Other caveats

- Covariate values used for weighting that were missing were imputed using hot-deck imputation. See the last last section for a report on the missingness of the data. It is worth noting that all individuals with missing Hispanic status were assigned to be non-Hispanic. Additionally, Hispanic status was used for the imputation of the race variable. Hispanic status was not used for the imputation of any other variable.
- All individuals who did not initially provide a sufficient quantity of blood were asked to provide a second specimen for testing. The subset of these individuals who did provide a second specimen had their value in the dataset for their test replaced by the test results for the second specimen. Thus, individuals who required two blood samples to have tests completed are treated identically to those who required only one blood sample. The 79 individuals who did not provide a second specimine were retained in the dataset and assigned a missing value for their test result.
- A small number (8) of the individuals who provided a second sample of blood were then included in the dataset twice. These individuals had two entries in the dataset used to construct the weights, this analysis. While these duplicates created small inaccuracies in the estimated weights that are used, we think these inaccuracies are ignorable.
- Two sequential tests were used to confirm seropositivity for each individual. To treat both tests as a single test, calculations were carried out to determine the sensitivity and specificity of the sequential testing procedure. This combination assumes that the tests are independent conditional on each individuals true status. A tool for this calculation and the formula used can be found here.
- The convenience sample had a higher proportion of individuals who thought they had previously been infected with COVID, and a higher proportion who had tested positive for COVID prior to this survey because of a suspected COVID illness.

### 1.4.1 Reporting Feeling Sick

```
make_sum_tabl <- function(var, valnames = c("Negative", "Positive", "Missing")) {
  outputFormat <- knitr::opts_knit$get("rmarkdown.pandoc.to")
  variable <- enexpr(var)
  sum_df <- surv_df %>% group_by(randsamp) %>% 
    summarise(Count = n(), 
              n_neg = sum(!!variable %in% 0),
              n_pos = sum(!!variable %in% 1), 
              n_mis = sum(is.na(!!variable)),
              percent_neg = round(100 * n_neg / Count, 2),
              percent_pos = round(100 * n_pos / Count, 2),
              percent_mis = round(100 * n_mis / Count, 2),
              pnm_neg = round(100 * n_neg / (n_neg + n_pos), 2),
              pnm_pos = round(100 * n_pos /(n_neg + n_pos), 2))
  
  sum_df$randsamp <- recode(sum_df$randsamp,
                            "n" = "Convenience", 
                            "y" = "Address-Based")
  names(sum_df)[1] <- "Sample Type"
  if (length(outputFormat) == 0) outputFormat <- "html"
  if (outputFormat %in% c('latex', 'html')) {
    sum_df %>% gt::gt() %>% gt::tab_spanner(
      label = "Number", 
      columns = vars(n_neg, n_pos, n_mis)
    ) %>% gt::tab_spanner(
      label = "Percentage", 
      columns = vars(percent_neg, percent_pos, percent_mis)
    ) %>%
      gt::tab_spanner(
        label = "Percentage of Non-Missing", 
        columns = vars(pnm_neg, pnm_pos)
      ) %>% gt::cols_label(
        n_neg = valnames[1], n_pos = valnames[2], n_mis = valnames[3],
        percent_neg = valnames[1], percent_pos = valnames[2],
        percent_mis = valnames[3], pnm_neg = valnames[1],
        pnm_pos = valnames[2], 
      )
  }else{
    sum_df %>% knitr::kable()
  }
}

make_sum_tabl(covid_illness, c("No", "Yes", "Missing"))
```

| Sample Type | Count | Number | | | Percentage | | | Percentage of Non-Missing | |
| --- | --- | --- | --- | --- | --- | --- | --- | --- | --- |
|  |  | No | Yes | Missing | No | Yes | Missing | No | Yes |
| Convenience | 504 | 295 | 90 | 119 | 58.53 | 17.86 | 23.61 | 76.62 | 23.38 |
| Address-Based | 860 | 615 | 110 | 135 | 71.51 | 12.79 | 15.70 | 84.83 | 15.17 |

### 1.4.2 Testing Results

```
make_sum_tabl(const_test_res)
```

| Sample Type | Count | Number | | | Percentage | | | Percentage of Non-Missing | |
| --- | --- | --- | --- | --- | --- | --- | --- | --- | --- |
|  |  | Negative | Positive | Missing | Negative | Positive | Missing | Negative | Positive |
| Convenience | 504 | 355 | 3 | 146 | 70.44 | 0.60 | 28.97 | 99.16 | 0.84 |
| Address-Based | 860 | 683 | 2 | 175 | 79.42 | 0.23 | 20.35 | 99.71 | 0.29 |

# 

**This issue is studied in greater detail in the senisitivy analysis section**

# 2 Implementation

## 2.1 Construction of the two-phase survey object

In the first stage, we construct a two-phase object that will account for the missing tests data due to “quantity not sufficient” of some individuals.

```
## This object is created for comparisons
surv_obj <- survey::svydesign(
  ids = ~HOUSEHOLD,
  weights = ~weight,
  strata = ~randsamp,
  data = surv_df %>% filter(!is.na(both_pos))
    )

two_phase_surv <- survey::twophase(
  id = list(~HOUSEHOLD, ~individual),
  strata = list(~randsamp, ~randsamp),
  weights = list(~weight, ~wt2),
  subset = ~I(qs), 
  data = surv_df, method = "approx"
)

make_pretty_out <- function(surv_obj) {
  sv_mean <- survey::svymean(~both_pos, surv_obj, na.rm = TRUE)
  est <- round(100 * as.numeric(sv_mean), 2)
  std_err <- round(100 * as.numeric(sv_mean %>% survey::SE()), 2)
  data.frame("Estimate" = est, "Standard Error" = std_err) 
}

make_table <- function(surv_objs, names) {
  outputFormat <- knitr::opts_knit$get("rmarkdown.pandoc.to")
  if ("survey.design" %in% class(surv_objs)) {
    fin_df <- make_pretty_out(surv_objs)
  } else {
    fin_df <- NULL
    for (sv_idx in seq_along(names)) {
      fin_df <- bind_rows(
        fin_df,
        bind_cols(
          "Survey Object" = names[sv_idx],
          make_pretty_out(surv_objs[[sv_idx]])
        )
      )
    }
  }
  if (length(outputFormat) > 0) {
    if (outputFormat %in% c('latex', 'html') ) { 
      fin_df %>% gt::gt() %>% gt::cols_label(
        "Standard.Error" = "Standard Error"
      ) %>% gt::tab_spanner(
    label = "Percent Positive (on both Tests)", 
    columns = c("Estimate", "Standard.Error")
  )
    }else{
      fin_df %>% knitr::kable()
    }
  }else{
    fin_df %>% gt::gt()
  }
}
```

### 2.1.1 Comparing estimates and standard errors

```
make_table(list(surv_obj, two_phase_surv), 
                c("Single-Phase Object (Dropping Missing Values)",
                  "Using Two-Phase Sampling"))
```

| Survey Object | Percent Positive (on both Tests) | |
| --- | --- | --- |
|  | Estimate | Standard Error |
| Single-Phase Object (Dropping Missing Values) | 3.3 | 0.83 |
| Using Two-Phase Sampling | 3.3 | 0.82 |

# 

Above, note that accounting for the missing values reduces the standard errors, but does not change the estimate.

## 2.2 Calibrating the two-phase object

The above code assumed that individuals were missing completely at random. However, we want to make a weaker assumption (missing at random). Thus, we call calibrate on the two-phase object to calibrate the second phase to the population. For more information (see section 9.2 of Lumley 2010):

```
post_cal_obj <- survey::calibrate(two_phase_surv, phase = 2,
   calfun = "raking", stage = 0, 
   ~AGECAT+HISPANIC+sex+RACECAT+INCOME)
```

### 2.2.1 Comparing estimates and standard errors

```
make_table(list(two_phase_surv, post_cal_obj), 
                c("Pre-Calibration", "Post-Calibration"))
```

| Survey Object | Percent Positive (on both Tests) | |
| --- | --- | --- |
|  | Estimate | Standard Error |
| Pre-Calibration | 3.30 | 0.82 |
| Post-Calibration | 3.22 | 0.81 |

**Note: This changes both the estimate and standard error**

## 2.3 Creating new single-phase object

Next we wish to perform raking on our weights from the first phase of our survey. Currently this is not supported with the two-phase object, so instead a new single-phase object is created with weights that approximately account for both of phases of the two-phase design.

It is worth noting that nothing methodological is happening in this step, **we are only creating a new object in R that will satisfy all of our needs.**

```
new_one_phase <- survey::svydesign(
  ids = ~HOUSEHOLD,
  weights = weights(post_cal_obj),
  strata = ~randsamp,
  data = subset(surv_df, qs == 1) 
)
```

Sanity Check

```
make_table(list(surv_obj, two_phase_surv$phase1$full, 
                two_phase_surv$phase1$sample, 
                post_cal_obj, new_one_phase), 
           c("Original single-phase object", 
             "Phase one object from two-phase object", 
             "Phase one object from two-phase object (ignoring missingness)", 
             "Previous two-phase object",
             "New single-phase object"))
```

| Survey Object | Percent Positive (on both Tests) | |
| --- | --- | --- |
|  | Estimate | Standard Error |
| Original single-phase object | 3.30 | 0.83 |
| Phase one object from two-phase object | 3.30 | 0.83 |
| Phase one object from two-phase object (ignoring missingness) | 3.30 | 0.83 |
| Previous two-phase object | 3.22 | 0.81 |
| New single-phase object | 3.22 | 0.80 |

## 2.4 Raking our survey object

Now that we have created a single phase object we can rake the survey object so that the fact the weights were calculated using raking will be accounted for in the estimation of standard errors. As a reminder, when weights are calculated using raking, standard errors of estimates using these weights are reduced because we are able to take into account properly the population information used for raking (Lumley 2010).

```
post_rake_obj <- survey::rake(
  new_one_phase,
  sample.margins = list(
    ~AGECAT + sex,
    ~HISPANIC + sex,
    ~RACECAT + sex,
    ~INCOME + sex
    # ~INCOME + hhs
  ),
  population.margins = list(
    ps_obj$age_sex[, c("AGECAT", "sex", "Freq")],
    ps_obj$hisp_sex[, c("HISPANIC", "sex", "Freq")],
    ps_obj$race_sex[, c("RACECAT", "sex", "Freq")],
    ps_obj$inc_sex[, c("INCOME", "sex", "Freq")]
    # ps_obj$inc_hhs[, c("INCOME", "hhs", "Freq")]
  )
)

my_summarise <- function(data, group_var) {
  data %>%
    group_by({{ group_var }}) %>%
    summarise(mean = mean(mass))
}
```

### 2.4.1 Comparing estimates and standard errors

```
make_table(list(new_one_phase, post_rake_obj), 
           c("Before Raking", "After Raking"))
```

| Survey Object | Percent Positive (on both Tests) | |
| --- | --- | --- |
|  | Estimate | Standard Error |
| Before Raking | 3.22 | 0.80 |
| After Raking | 3.24 | 0.73 |

Note above that the standard error has been reduced. **In normal circumstances, we would expect there to be no change in the estimate, but since we have eliminated 8 observations that were duplicates since the original weights were calculated, the estimate changes by a small amount.**

## 2.5 Accounting for sensitivity and specificity:

While all of the above estimates were providing estimates for the proportion of individuals who tested positive on both tests, we are actually interested in estimating prevalence or the proportion of individuals who have been infected with COVID19.

One complication for this correction is that sero-positivity was determined using a sequence of two tests. A first test was conducted, and individuals who tested positive on the first test were given a second test. Only individuals who tested positive on both tests were counted as positive. This combination assumes that the tests are independent conditional on each individuals true status, and allows us to treat this sequential testing procedure as a single test. The calculations used for this combination can be found on the epitools website “Epitools - Sensitivity and Specificity of Two Tests Used ...” (n.d.). Note that the sensitivity and specificity of these tests are derived using positive and negative predictive agreement respectively with other benchmark tests. For more details on the comparators used for the Ab and ElisaG tests, see “Platelia SARS-CoV-2 Total Ab” (n.d.) and “Anti-SARS-CoV-2 ELISA (IgG) Instruction for Use” (n.d.) respectively.

```
combine_tests <- function(t1_spec, t1_sens, t2_spec, t2_sens) {
  return(c(
    "spec" = 1 - (1 - t1_spec) * (1 - t2_spec),
    "sens" = t1_sens * t2_sens
  )
  )
}

## First (AB) test
## Combined results
## Positive Percent Agreement: 92.16% (47/51); 95% CI: (81.5% - 96.91%) 
## Negative Percent Agreement: 99.56% (684/687); 95% CI: (98.72 – 99.85%)
## Second Test: Anti-SARS-CoV-2 ELISA (IgG)
#IgG Sensitivity (PPA) 90% (27/30) (74.4%; 96.5%) (midpoint: 85.45)
#IgG Specificity (NPA) 100% (80/80) (95.4%; 100%) (midpoint: 97.7)

testing_accuracy <- data.frame(
  "Test" = c("Bio-Rad Ab test", "Euroimmun elisaG (IgG)"),
  "Specificity" = c(0.9956, 1),
  "Sensitivity" = c(0.9216, 0.9)
)

comb_sens_spec <- combine_tests(
  t1_spec = testing_accuracy$Specificity[1],
  t1_sens = testing_accuracy$Sensitivity[1],
  t2_spec = testing_accuracy$Specificity[2],
  t2_sens = testing_accuracy$Sensitivity[2]
)

tabl_dff <- testing_accuracy %>% mutate(
  Specificity = paste0(100 * Specificity, "%"),
  Sensitivity = paste0(100 * Sensitivity, "%"),
  ) %>% bind_rows(
    c("Test" = "Sequential Test", 
      "Specificity" = paste0(round(100 * comb_sens_spec["spec"], 2), "%"),
      "Sensitivity" = paste0(round(100 * comb_sens_spec["sens"], 2), "%"))
  )
colnames(tabl_dff)[2:3] <- c("Negative Percent agreement",
                             "Positive percent agreement")
gt::gt(
  tabl_dff
) %>% gt::cols_align(
  align = "center",
  columns = 2:3
)
```

| Test | Negative Percent agreement | Positive percent agreement |
| --- | --- | --- |
| Bio-Rad Ab test | 99.56% | 92.16% |
| Euroimmun elisaG (IgG) | 100% | 90% |
| Sequential Test | 100% | 82.94% |

The estimates of sensitivity and specificity used come from reports of the tests’ performance. The source for the Ab test can be found below the last table of page 15 of “Platelia SARS-CoV-2 Total Ab” (n.d.) (link to document). The source for the IgG test can be found in the last table of page 12 of “Anti-SARS-CoV-2 ELISA (IgG) Instruction for Use” (n.d.)(link to document).

Next, recall the formula that relates the proportion of individuals with positive tests to the proportion of individuals who have been infected with COVID19:

\[
\text{Prevalence} = \frac{\text{Proportion Positive} + \text{Specificity} - 1 }{\text{Sensitivity} + \text{Specificity} - 1}
\]

While there is a single agreed upon method for adjusting the observed proportion of positive tests to obtain an estimate of prevalence, there is no such analog for the standard error.

Here, we draw from a suggestion by REICZIGEL, FÖLDI, and ÓZSVÁRI (2010) for how to adjust our confidence limits in the presence of a test with known sensitivity and specificity.

- This method was described for settings in which all observations are independent and identically distributed. While this is not the case in our setting, by calculating the design effect of our study, we can calculate the **effective sample size** of our study, and the corresponding number of positive results that would be observed in this (smaller) population.
- The **effective sample size** is the sample size of an “equivalent” study in which all data are independent and identically distributed. Here “equivalent” means having the same estimates and standard errors.

The adjustment described by REICZIGEL, FÖLDI, and ÓZSVÁRI (2010) is based on inverting a hypothesis test. At a high level, this is done using the following method:

- For a proposed number of positive individuals \(x\), it is possible to estimate the probability that **exactly** this many individuals were truly positive given the observed number of positive tests and the sensitivity and specificity of the test.
- Once this value is calculated for every possible \(x\), a distribution can be constructed for all possible values of the prevalence (by dividing \(x\) by the sample size).
- Next, an interval is selected such that 95% of the distributions mass is contained within this interval (and contains the estimate near the middle).
- **Note that this has been implemented in an R function posted on the authors’ website. The code for this function can be seen by opening the “Code to adjust confidence limits” tab below.**

Code to adjust confidence limits

```
ci4prev=function(poz,n,se=1,sp=1,level=.95,dec=3,method="bl"){
  # calculates exact confidence limits for the prevalence of disease
  # adjusted for sensitivity and specificity of the diagnostic test
  
  # written by Jenő Reiczigel, 2009 (reiczigel.jeno@aotk.szie.hu)
  
  # if using please cite 
  #  Reiczigel, Földi and Ózsvári (2010) Exact confidence limits for 
  #  prevalence of a disease with an imperfect diagnostic test, 
  #  Epidemiology and infection, 138: 1674-1678.
  
  # poz - number of test positives
  # n - sample size
  # se - test sensitivity
  # sp - test specificity
  # level - prescribed confidence level
  # dec - required number of decimals for the result
  # method - "bl" for Blaker, "st" for Sterne, 
  #          "cp" for Clopper-Pearson, "wi" for Wilson (for n>500)
  
  # ci4prev calls the following functions:
  #     blakerci()  for Blaker's interval (see below), 
  #     sterne.int()  for Sterne's interval (see below),
  #     binconf()  from library(Hmisc) for Wilson & Clopper-Pearson
  
  if(method=="cp") cl=binconf(poz,n,method="e",alpha=1-level)[2:3]
  else if (method=="st") cl=sterne.int(poz,n,alpha=1-level)
  else if (method=="bl") cl=blakerci(poz,n,level=level)
  else if (method=="wi") cl=binconf(poz,n,method="w",alpha=1-level)[2:3]
  else stop('valid methods are "bl", "st", "cp", and "wi"')
  
  adj.cl=(cl+sp-1)/(se+sp-1) 
  adj.cl=pmax(adj.cl,c(0,0))
  adj.cl=pmin(adj.cl,c(1,1))
  
  names(adj.cl)=NULL
  return(round(adj.cl,digits=dec))
}

require(Hmisc)
# -----------------------------------
# Blaker's interval (by Helge Blaker)
# -----------------------------------

blakerci <- function(x,n,level=.95,tolerance=1e-04){
  lower = 0
  upper = 1
  if (x!=0){lower = qbeta((1-level)/2, x, n-x+1)
  while (acceptbin(x, n, lower + tolerance) < (1 - level))
    lower = lower+tolerance
  }
  if (x!=n){upper = qbeta(1 - (1-level)/2, x+1, n-x)
  while (acceptbin(x, n, upper - tolerance) < (1 - level))
    upper = upper-tolerance
  }
  c(lower,upper)
}
# Computes the Blaker exact ci (Canadian J. Stat 2000)
# for a binomial success probability
# for x successes out of n trials with
# confidence coefficient = level; uses acceptbin function

acceptbin = function(x, n, p){
  #computes the Blaker acceptability of p when x is observed
  # and X is bin(n, p)
  p1 = 1 - pbinom(x - 1, n, p)
  p2 = pbinom(x, n, p)
  a1 = p1 + pbinom(qbinom(p1, n, p) - 1, n, p)
  a2 = p2 + 1 - pbinom(qbinom(1 - p2, n, p), n, p)
  return(min(a1,a2))
}

######################################################################
#
# EXACT CONFIDENCE BOUNDS FOR A BINOMIAL PARAMETER p
# ~~~~~~~~~~~~~~~~~~~~~~~~~~~~~~~~~~~~~~~~~~~~~~~~~~
#
# Calculate exact Sterne confidence bounds for a binomial parameter
# by the method described in Duembgen (2004): Exact confidence
# bounds in discrete models - algorithmic aspects of Sternes method.
# Preprint, available on www.stat.unibe.ch/~duembgen
#
# Kaspar Rufibach, August 2004
#
######################################################################

# define function sterne.int (find example at the bottom)
sterne.int <- function(x,n,alpha=0.05,del=10^-5){
  
  logit <- function(p){log(p/(1-p))}
  invlogit <- function(y){exp(y)/(1+exp(y))}
  theta <- function(k,x,n){(lchoose(n,x)-lchoose(n,k))/(k-x)}
  Feta <- function(x,eta){pbinom(x,n,invlogit(eta))}
  
  ##############################################################
  # The function pi_eta(X,eta) automatically accounts for the
  # fact that if k_alpha(X)=min(J) then a_alpha^st(X)=a_alpha(X)
  
  piXeta <- function(x,eta){
    if (invlogit(eta)>=1){f <- 0} else {
      J <- c(0:(x-1),(x+1):n)
      
      # on (-infty,theta_0]
      t1 <- theta(0,x,n)
      if (is.na(t1)!=1 && eta<=t1){f <- 1-Feta(x-1,eta)}
      
      # on [theta_0,mode]
      k1 <- J[J<(x-1)]
      if (length(k1)>0){
        the1 <- theta(k1,x,n)
        the2 <- theta(k1+1,x,n)
        pos <- (the1<=eta)*(eta<the2)
        if (sum(pos)>0){f <- 1-Feta(x-1,eta)+Feta(max(k1*pos),eta)}}
      
      # mode
      the1 <- theta(x-1,x,n)
      the2 <- theta(x+1,x,n)
      if (eta>=the1 && eta<=the2){f <- 1}}
    
    # on [mode,theta_n]
    k2 <- J[J>(x+1)]
    if (length(k2)>0){
      the1 <- theta(k2-1,x,n)
      the2 <- theta(k2,x,n)
      kre <- sum(k2*(the1<eta)*(eta<=the2))
      if (kre>0){f <- 1-Feta(kre-1,eta)+Feta(x,eta)}}
    
    # on [theta_n,infty)
    t2 <- theta(n,x,n)
    if (is.na(t2)!=1 && eta>=t2){f <- Feta(x,eta)}
    f}
  
  #####################################
  # lower bound a_alpha^st(X)
  if (x==0){pu <- 0} else {
    J <- c(0:(x-1),(x+1):n)
    k1 <- min(J)
    pi1 <- piXeta(x,theta(k1,x,n))
    
    # calculation of k_alpha(X)
    if (pi1>=alpha){kal <- k1} else {
      k <- x-1
      while (k1<k-1){
        k2 <- floor((k+k1)/2)
        pi2 <- piXeta(x,theta(k2,x,n))
        if (pi2>=alpha){k <- k2} else {k1 <- k2}
      }
      kal <- k
    }
    
    # calculation of a_alpha^st(X)
    b1 <- theta(kal,x,n)
    pi1 <- 1-Feta(x-1,b1)+Feta(kal-1,b1)
    if (pi1<=alpha){b <- b1} else {
      b <- max(theta(kal-1,x,n),logit(del))
      pi <- 1-Feta(x-1,b)+Feta(kal-1,b)
      while (b1-b>del || pi1-pi>del){
        b2 <- (b+b1)/2
        pi2 <- 1-Feta(x-1,b2)+Feta(kal-1,b2)
        if (pi2>alpha){
          b1 <- b2
          pi1 <- pi2} else {
            b <- b2
            pi <- pi2}}}
    pu <- invlogit(b)}
  
  ######################################
  # upper bound b_alpha^st(X)
  if (x==n){po <- 1} else {
    J <- c(0:(x-1),(x+1):n)
    k1 <- max(J)
    pi1 <- piXeta(x,theta(k1,x,n))
    
    # calculation of k_alpha(X)
    if (pi1>=alpha){kau <- k1} else {
      k <- x+1
      pi <- 1
      while (k1>k+1){
        k2 <- floor((k+k1)/2)
        pi2 <- piXeta(x,theta(k2,x,n))
        if (pi2>=alpha){k <- k2} else {k1 <- k2}
      }
      kau <- k
    }
    
    # calculation of b_alpha^st(X)
    b1 <- theta(kau,x,n)
    pi1 <- 1-Feta(kau,b1)+Feta(x,b1)
    
    if (pi1<=alpha){
      b <- b1
      po <- pi1} else {
        b <- min(theta(kau+1,x,n),b1+n)
        pi <- 1-Feta(kau,b)+Feta(x,b)
        while (b-b1>del || pi1-pi>del){
          b2 <- (b+b1)/2
          pi2 <- 1-Feta(kau,b2)+Feta(x,b2)
          if (pi2>alpha){
            b1 <- b2
            pi1 <- pi2} else {
              b <- b2
              pi <- pi2}}}
    po <- invlogit(b)}
  
  c('a_alpha^St'=pu,'b_alpha^St'=po)
}
```

```
adj_for_sesp <- function(AP, Sp, Se) { (AP + Sp - 1) / (Se + Sp - 1) }
post_adj_est <- function(survey_obj, se = comb_sens_spec["sens"],
                         sp = comb_sens_spec["spec"], digs = 2){
  srvy_mean <-survey::svymean(~both_pos, survey_obj, 
                          deff = "replace")
  prop_pos <- as.numeric(srvy_mean)
  if (is.null(survey_obj$variables)) {
    survey_obj$variables <- survey_obj$phase1$sample$variables
  }
  iid_n <- nrow(survey_obj$variables) / survey::deff(srvy_mean)
  prev_est <- round(100 * adj_for_sesp(prop_pos, sp, se), digs)
  num_pos <- round(iid_n * prop_pos)
  ci <- ci4prev(num_pos, round(iid_n), se = se, sp = sp, dec = 2 + digs)
  chrs <- paste0("%.", digs, "f")
  tibble(
    # "Effective Sample Size" = iid_n,
    # "Number Tested Positive" = num_pos,
    "Prevalence Estimate (95% CI)" = sprintf(paste0(chrs, " (", 
                                                    chrs, ", ",chrs, ")"),
                                             prev_est, 100 * ci[1], 
                                             100 * ci[2]))
}

prop_pos_by <- function(survey_obj, form, revalkey = NULL,
                        se = comb_sens_spec["sens"], 
                        sp = comb_sens_spec["spec"],
                        digs = 4, return_type = "tab", 
                        oth_col_name = NULL){
  prop_pos <- survey::svyby(~both_pos, form,
                            survey_obj, survey::svymean, 
                            deff = "replacem")
  vname <- colnames(prop_pos)[1]
  prop_pos[, 1] <- as.character(prop_pos[, 1])
  adj_df <- prop_pos %>% mutate(across(.cols = c("both_pos", "se"), 
                                       .fns = function(x) round(x * 100, 2)))
  adj_df$prev_est <- round(adj_for_sesp(adj_df$both_pos, 
                                        Sp = sp, Se = se), digs)
  counts <- surv_obj$variables %>%
    group_by(eval(parse(text = vname))) %>%
    count()
  colnames(counts)[1] <- vname
  counts[[vname]] <- as.character(counts[[vname]])
  efss <- left_join(adj_df, counts, by = vname) %>% 
    mutate(efs = n / DEff.both_pos, 
           num_pos = round(efs * prev_est / 100), 
           efs = round(efs))
  efss$ub <- efss$lb <- NA
  for (bound_idx in 1:nrow(efss)) {
    if (!is.na(efss$num_pos[bound_idx])) {
      efss[bound_idx, c("lb", "ub")] <- 
      round(100 * ci4prev(efss$num_pos[bound_idx],
                          efss$efs[bound_idx], se = se,
                          sp = sp, dec = 2 + digs), digs)
    }else{
      efss[bound_idx, c("prev_est", "lb", "ub")] <- NA 
    }
  }
  if (return_type == "tab") {
    adj_df <- efss %>% mutate(
      est_ci = sprintf("%.2f (%.2f, %.2f)",
                       prev_est, lb, ub)
    ) %>% select(1, both_pos, se, est_ci)
    if (!is.null(revalkey)) {
      adj_df[, 1] <- dplyr::recode(
        adj_df[, 1],  !!!revalkey
      )
    } 
    if (!is.null(oth_col_name)) {
      colnames(adj_df)[1] <- oth_col_name
    }
    adj_df %>% gt::gt() %>% gt::tab_spanner(
      label = "Percent Positive (on both Tests)", 
      columns = c("both_pos", "se")
    ) %>% gt::tab_spanner(
      label = "Prevalence", 
      columns = c("est_ci")
    ) %>%
      gt::cols_label(
        "both_pos" = "Estimate",
        "se" = "Standard Error",
        "est_ci" = "Estimate (95% CI)"
      ) %>% gt::cols_align("center")
  }else{
    efss
  }
}

source("bootstrap_ci.R")
desn_eff <- survey::svymean(~both_pos, post_rake_obj, deff = "replace") %>%  survey::deff()
fin_reslt <- post_adj_est(survey_obj = post_rake_obj, digs = 2) %>% gt::gt() %>%
  gt::cols_align("center") %>% gt::tab_footnote(
    footnote = paste0("The effective sample size is ",
                      round(nrow(post_rake_obj$variables) / desn_eff), 
                      " for these calculations."),
    locations = gt::cells_column_labels(
      columns = vars(`Prevalence Estimate (95% CI)`))
  )

fin_reslt
```

| Prevalence Estimate (95% CI)1 |
| --- |
| 3.91 (2.35, 5.96) |
|  |
| --- |
| *1* The effective sample size is 589 for these calculations. |

## 2.6 Subgroup Analysis

All subgroup specific estimates are calculated using the all the adjustments described above.

### 2.6.1 Race

#### 2.6.1.1 Table

```
revalkey <- c("1" = "White", "2" = "Black", "3" = "Asian", "4" = "Other")
prop_pos_by(post_rake_obj, ~RACECAT, revalkey, oth_col_name = "Race")
```

| Race | Percent Positive (on both Tests) | | Prevalence |
| --- | --- | --- | --- |
|  | Estimate | Standard Error | Estimate (95% CI) |
| White | 1.44 | 0.47 | 1.74 (1.02, 3.57) |
| Black | 9.77 | 3.41 | 11.78 (7.25, 24.77) |
| Asian | 1.95 | 1.11 | 2.35 (1.05, 7.41) |
| Other | 10.75 | 4.77 | 12.96 (7.53, 33.10) |

#### 2.6.1.2 Plot

```
plot_df <- prop_pos_by(post_rake_obj, ~RACECAT, 
                       revalkey, return_type = "data")
plot_df$RACECAT <- recode(plot_df$RACECAT, !!!revalkey)

ggplot(plot_df, aes(x = RACECAT, y = prev_est / 100,
                    ymin = lb / 100, ymax = ub/100)) + 
  geom_point(size = 1) + geom_pointrange(size = 1) + 
  ylim(0, NA) +
  ylab("Estimated Prevalence") + 
  labs(title = "Estimated Prevalence Across Race Group",
       subtitle = "In King County in early to mid August 2020",
       caption = "Points indicate estimates and lines indicate 95% CI") +
  # scale_y_log10(labels = scales::percent) +
  scale_y_continuous(labels = scales::percent, limits = c(0, NA)) +
  coord_flip() + theme(
    axis.title.y = element_blank(),
    axis.title.x = element_blank()
  )
```

### 2.6.2 Sex

#### 2.6.2.1 Table

```
sexkey <- c("1" = "Male", "2" = "Female")
prop_pos_by(post_rake_obj, ~sex, sexkey, oth_col_name = "Sex")
```

| Sex | Percent Positive (on both Tests) | | Prevalence |
| --- | --- | --- | --- |
|  | Estimate | Standard Error | Estimate (95% CI) |
| Male | 3.64 | 1.03 | 4.39 (2.99, 8.50) |
| Female | 2.84 | 0.91 | 3.42 (2.37, 7.38) |

#### 2.6.2.2 Plot

```
plot_df_sex <- prop_pos_by(post_rake_obj, ~sex, 
                       revalkey, return_type = "data")
plot_df_sex$sex <- recode(plot_df_sex$sex, !!!sexkey)
ggplot(plot_df_sex, aes(x = sex, y = prev_est / 100,
                        ymin = lb / 100, ymax = ub / 100)) + 
  geom_pointrange(size = 1) + 
  ylim(0, NA) +
  ylab("Estimated Prevalence") + 
  labs(title = "Estimated Prevalence Across Sex",
       subtitle = "In King County in early to mid August 2020",
       caption = "Points indicate estimates and lines indicate 95% CI") +
  # scale_y_log10(labels = scales::percent) +
  scale_y_continuous(labels = scales::percent, limits = c(0, NA)) +
  coord_flip() + theme(
    axis.title.y = element_blank(),
    axis.title.x = element_blank()
  )
```

### 2.6.3 Income

#### 2.6.3.1 Table

```
prop_pos_by(post_rake_obj, ~binary_income, oth_col_name = "Income") %>%
   gt::tab_footnote( 
  footnote = "Median household income in King County is $95,000",
    locations = gt::cells_column_labels(
      columns = vars(Income)))
```

| Income1 | Percent Positive (on both Tests) | | Prevalence |
| --- | --- | --- | --- |
|  | Estimate | Standard Error | Estimate (95% CI) |
| Above 100,000 | 1.30 | 0.47 | 1.57 (0.94, 3.48) |
| At or below 100,000 | 6.09 | 1.66 | 7.34 (4.96, 13.99) |
|  |  |  |  |
| --- | --- | --- | --- |
| *1* Median household income in King County is $95,000 | | | |

#### 2.6.3.2 Plot

```
inc_df <- prop_pos_by(post_rake_obj, ~binary_income, return_type = "data")
ggplot(inc_df, aes(x = binary_income, y = prev_est / 100,
                   ymin = lb / 100, ymax = ub / 100)) + 
  geom_pointrange(size = 1) + 
  ylab("Estimated Prevalence") + 
  labs(title = "Estimated Prevalence Across Income Categories",
       subtitle = "In King County in early to mid August 2020",
       caption = "Points indicate estimates and lines indicate 95% CI") + 
  # scale_y_log10(labels = scales::percent) +
  scale_y_continuous(labels = scales::percent, limits = c(0, NA)) +
  coord_flip() + theme(
    axis.title.y = element_blank(),
    axis.title.x = element_blank()
  )
```

### 2.6.4 Age Group

#### 2.6.4.1 Table

```
prop_pos_by(post_rake_obj, ~agegrp2, oth_col_name = "Age Group")
```

| Age Group | Percent Positive (on both Tests) | | Prevalence |
| --- | --- | --- | --- |
|  | Estimate | Standard Error | Estimate (95% CI) |
| 0≤age<16 | 2.10 | 1.19 | 2.53 (1.12, 7.98) |
| 16≤age<25 | 9.05 | 4.85 | 10.91 (4.81, 31.97) |
| 25≤age<45 | 1.83 | 0.71 | 2.21 (1.19, 5.25) |
| 45≤age<65 | 4.04 | 1.47 | 4.87 (3.03, 11.14) |
| 65≤age<100 | 2.50 | 1.40 | 3.01 (1.32, 9.31) |

#### 2.6.4.2 Plot

```
agrp_df <- prop_pos_by(post_rake_obj, ~agegrp2, return_type = "data",
            oth_col_name = "Age Group")
ggplot(agrp_df, aes(x = agegrp2, y = prev_est / 100,
                    ymin = lb / 100, ymax = ub / 100)) + 
  geom_pointrange(size = 1) + 
  ylab("Estimated Prevalence") + 
  labs(title = "Estimated Prevalence Across Age Group",
       subtitle = "In King County in early to mid August 2020",
       caption = "Points indicate estimates and lines indicate 95% CI") + 
  # scale_y_log10(labels = scales::percent) + 
  scale_y_continuous(labels = scales::percent, limits = c(0, NA)) +
  coord_flip() + theme(
    axis.title.y = element_blank(),
    axis.title.x = element_blank()
  )
```

## 2.7 Between Group Testing

In this section, we consider testing for between group difference across the previously considered categorical variables (race, sex, income, and age group). For these comparisons, we consider pairwise comparisons between different levels of a categorical variable as part of a single question. However we treat hypotheses concerned with different categorical variables as distinct from one another. As an example we consider the comparison between Blacks and whites and the comparison between Blacks and Asians as part of a single question, but consider the comparison between Blacks and Asians separate from the comparison between males and females. Thus, we adjust for multiple comparisons between levels within categories, but not for the multiple categorical variables. We control the family-wise error rate (FWER) using a Bonferroni adjustment for pairwise comparisons between different groups within each category, but do not adjust for the number of categorical variables for which we are making between-category comparisons (in our case four).

Each table below displays the Bonferroni-corrected p-value for each possible pairwise comparison.

### 2.7.1 Race

```
make_resl <- function(var, pval_adj = TRUE) {
  bval <- unique(new_one_phase$variables[[var]])
  bval <- bval[!is.na(bval)]
  if (!is.numeric(bval)) {
    bval <- paste0("'", bval, "'")
    repl_str <- ","
  }else{
    repl_str <- ",|\\ "
  }
  all_res <- NULL
  for (bv_idx in seq_along(bval)) {
    sub_res <- survey::svyglm(
      as.formula(paste0("both_pos ~ relevel(as.factor(",
                        var, "), ", bval[bv_idx],  ")")),
      family = "binomial",
      new_one_phase) %>% summary() %>% coef()
    levels <- map(str_split(rownames(sub_res), "\\)"), 
        function(x) {
          y <- x[length(x) - c(1, 0)]
          gsub(repl_str, "", y)
          })[-1]
    levels <- do.call(rbind, levels)
    sub_res <- data.frame(
      level1 = levels[, 1], level2 = levels[, 2],
      odds_ratio = exp(sub_res[-1, 1]),
      inv_confint = paste0(
        "(", round(1 / exp(sub_res[-1, 1] + qnorm(0.975) * sub_res[-1, 2]), 1), 
        ", ", round(1 / exp(sub_res[-1, 1] - qnorm(0.975) * sub_res[-1, 2]), 1),
        ")"),
      pval = sub_res[-1, ncol(sub_res)]
    ) 
    rownames(sub_res) <- NULL
    all_res <- bind_rows(sub_res, all_res)
  }
  if (pval_adj) {
    return(all_res %>% mutate(
      pval = as.character(round(pmin(1, pval * nrow(all_res) / 2), 6))
    ) )
  } else {
    return(all_res)
  }
}

table_pairwise <- function(pval_tab, rename_vec) {
  pval_tab$odds_ratio <- pval_tab$inv_confint <- NULL
  pval_tab$level1 <- gsub("\\\"|\\ ", "", pval_tab$level1)
  if (!is.null(rename_vec)) {
    rfrmt <- pval_tab %>% mutate(
    level1 = recode(level1, !!!rename_vec),
    level2 = recode(level2, !!!rename_vec))
  }else{
    rfrmt <- pval_tab 
  }
  rfrmt <- rfrmt %>% arrange(level1, level2)
  rfrmt <- rfrmt %>% 
    pivot_wider(names_from = level2, values_from = pval,
                    values_fill = "") %>%
    arrange(level1)
  rfrmt <- rfrmt[, c(1, 1 + order(colnames(rfrmt)[-1]))]
  rfrmt %>% gt::gt(rowname_col = "level1") %>%
    gt::tab_spanner("Comparison Group", 2:ncol(rfrmt)) %>%
    gt::cols_align("center") %>% gt::tab_stubhead(label = "Baseline Group")
}

race_rename <- c("1" = "White", "2" = "Black",
                 "3" = "Asian", "4" = "Other")
table_pairwise(make_resl("RACECAT"), race_rename)
```

| Baseline Group | Comparison Group | | | |
| --- | --- | --- | --- | --- |
|  | Asian | Black | Other | White |
| Asian |  | 0.101053 | 0.132775 | 1 |
| Black | 0.101053 |  | 1 | 0.000978 |
| Other | 0.132775 | 1 |  | 0.003113 |
| White | 1 | 0.000978 | 0.003113 |  |

### 2.7.2 Sex

```
sex_rename <- c("1" = "Male", "2" = "Female")
table_pairwise(make_resl("sex"), sex_rename)
```

| Baseline Group | Comparison Group | |
| --- | --- | --- |
|  | Female | Male |
| Female |  | 0.550988 |
| Male | 0.550988 |  |

### 2.7.3 Income

```
table_pairwise(make_resl("binary_income"), NULL)
```

| Baseline Group | Comparison Group | |
| --- | --- | --- |
|  | Above 100000 | At or below 100000 |
| Above100000 |  | 0.000984 |
| Atorbelow100000 | 0.000984 |  |

### 2.7.4 Age Group

```
table_pairwise(make_resl("agegrp2"), NULL)
```

| Baseline Group | Comparison Group | | | | |
| --- | --- | --- | --- | --- | --- |
|  | 0≤age<16 | 16≤age<25 | 25≤age<45 | 45≤age<65 | 65≤age<100 |
| 0≤age<16 |  | 0.767777 | 1 | 1 | 1 |
| 16≤age<25 | 0.767777 |  | 0.156947 | 1 | 1 |
| 25≤age<45 | 1 | 0.156947 |  | 1 | 1 |
| 45≤age<65 | 1 | 1 | 1 |  | 1 |
| 65≤age<100 | 1 | 1 | 1 | 1 |  |

## 

After considering all pairwise differences between groups, the following groups were found to be significantly different at the \(0.05\) level:

```
signf <- bind_rows(
  make_resl("RACECAT") %>% mutate(
    level1 = recode(level1, !!!race_rename),
    level2 = recode(level2, !!!race_rename), 
    Category = "Race"),
  make_resl("sex") %>% mutate(
    level1 = recode(level1, !!!sex_rename),
    level2 = recode(level2, !!!sex_rename), 
    Category = "Sex"),
  make_resl("binary_income") %>% mutate(Category = "Income"),
  make_resl("agegrp2") %>% mutate(Category = "Age Group")) %>%
  filter(pval < 0.05) %>% group_by(pval) %>%
  filter(row_number() == 1) %>% ungroup()
signf$level1 <- gsub("\\\"", "", signf$level1)
signf <- signf %>% mutate(pval = round(as.numeric(pval), 6))
signf$odds_ratio <- round(1 / signf$odds_ratio, 1)
gt::gt(signf[, c(2, 1, 3, 4, 5, 6)] %>% group_by(Category) ) %>%
gt::cols_label(level2 = gt::html("Baseline <br> Group"),
               level1 = gt::html("Comparison <br> Group"), 
               odds_ratio = gt::html("Estimated <br> Odds Ratio"), 
               inv_confint = gt::html("Confidence <br> Interval"), 
               pval = gt::html("Bonferroni-Adjusted <br> P-value")) %>%
 gt::cols_align(columns = 1:5, align = "c")
```

| Baseline   Group | Comparison   Group | Estimated   Odds Ratio | Confidence   Interval | Bonferroni-Adjusted   P-value |
| --- | --- | --- | --- | --- |
| Race | | | | |
| White | Other | 8.2 | (2.5, 26.9) | 0.003113 |
| White | Black | 7.3 | (2.6, 20.6) | 0.000978 |
| Income | | | | |
| Above 100000 | At or below 100000 | 5.0 | (1.9, 12.8) | 0.000984 |

```
## checking Banjamini-Hochberg method
all_res <- list(make_resl("RACECAT", pval_adj = FALSE), 
                     make_resl("sex", pval_adj = FALSE),
                     make_resl("binary_income", pval_adj = FALSE),
                     make_resl("agegrp2", pval_adj = FALSE))
# install.packages("sgof")
make_pval_mat <- function(x){
  look <- all_res[[x]] %>%
    mutate(pval = round(pval, 9)) %>%
    group_by(pval) %>% 
    filter(row_number() == 1)
  fin_df <- round(cbind(pvalue = sort(look$pval),
      cutoff_Bonf = 0.005 / length(look$pval),
      cutoff_BH = 0.005 * seq(look$pval) / length(look$pval),
      cutoff_BY = 0.005 * seq(look$pval) / (length(look$pval) * 
                                             sum(1 / seq(look$pval)))), 5)
  cbind(fin_df,
        "rej_Bonf" = fin_df[, 1] < fin_df[, 2],
        "rej_BH" = fin_df[, 1] < fin_df[, 3],
        "rej_BY" = fin_df[, 1] < fin_df[, 4])
}
# map(1:4, make_pval_mat)
```

# 3 Sensitivity Analysis

## 3.1 Sample rebalancing to account for biased convenience sample

As mentioned earlier, the convenience sample is likely to be biased towards having more COVID19 positive individuals. One solution to account for this would be to adjust weights based on those in the sample who reported testing positive on a previous COVID19 test. Unfortunately, the openly available data from King County do not match the collected data in the following ways:

- Individuals completing the survey were first asked if they felt ill with COVID in the past. Conditional on answering yes to this question, they were then asked if they had gotten tested, and if they tested what was the test result. Individuals in the survey were not asked how many times they were tested for COVID, or how many times they tested positive. Additionally, many individuals did not respond to these questions.
- The data for King county would allow for the calculation of the cumulative number of tests (both positive and negative). However, identifying the number of **unique** individuals who had tested positive after reporting to feel ill from COVID is not available. Last, the King county testing statistics include all tests, not just those motivated by feeling ill.

As an alternative to using King County data, we treat the address-based sample and the given survey weights to construct a distribution of those with positive tests. We then use this distribution to adjust the convenience sample weights such that the weighted distribution of positive tests in both strata (address-based and convenience sample) match. This could have been used in our main analysis, but there were multiple issues with this approach:

- While it is possible to make such adjustments to the weights, this procedure is not well understood. Thus it would be difficult to properly account for the uncertainty introduced by make this adjustment.
- There were many missing values for the survey responses, which could also result in unaccounted for variability.

More statistically principled solutions to account for bias in convenience based samples exist (see Valliant 2020), and would be worth considering given more time. These alternative approaches would likely require re-calculation of the weights for the entire sample.

```
## Post-stratify on the observed number of positive tests
## in the address-based sample
make_ps_est <- function(svy_df, des_obj, type = "none") {
  surv_df <- svy_df
  
  props <- surv_df %>% group_by(randsamp) %>% 
    summarise(prop_felt_sick = mean(covid_illness, na.rm = TRUE),
              prop_test_pos = mean(const_test_res, na.rm = TRUE))
  w_mis_il_conv <- 
    which(surv_df$randsamp == "n" & is.na(surv_df$covid_illness))
  surv_df[w_mis_il_conv, "covid_illness"] <-
    rbinom(length(w_mis_il_conv), size = 1, 
           prob = props %>% filter(randsamp == "n") %>% 
             pull(prop_felt_sick))
  w_mis_il_abs <- 
    which(surv_df$randsamp == "y" & is.na(surv_df$covid_illness))
  surv_df[w_mis_il_abs, "covid_illness"] <-
    rbinom(length(w_mis_il_abs), size = 1, 
           prob = props %>% filter(randsamp == "y") %>% 
             pull(prop_test_pos))
  w_mis_pt_conv <- 
    which(surv_df$randsamp == "n" & is.na(surv_df$const_test_res))
  surv_df[w_mis_pt_conv, "const_test_res"] <-
    rbinom(length(w_mis_pt_conv), size = 1, 
           prob = props %>% filter(randsamp == "n") %>% 
             pull(prop_test_pos))
  w_mis_pt_abs <- 
    which(surv_df$randsamp == "y" & is.na(surv_df$const_test_res))
  surv_df[w_mis_pt_abs, "const_test_res"] <-
    rbinom(length(w_mis_pt_abs), size = 1, 
           prob = props %>% filter(randsamp == "y") %>% 
             pull(prop_test_pos))
  
  ps_one_phase <- survey::svydesign(
    ids = ~HOUSEHOLD,
    weights = weights(post_cal_obj),
    strata = ~randsamp,
    data = subset(surv_df, qs == 1) 
  )  
  if (type == "pos_test") {
    pos_tests <- 
      surv_df %>% filter(randsamp == "y") %>% 
      group_by(const_test_res) %>% count(name = "Freq")
    
    post_strat_obj <- survey::postStratify(
      ps_one_phase, ~const_test_res, pos_tests
    )
  }else if (type == "cov_ill") {
  ## Post-stratify on the number of individuals reporting
  ## having felt sick with COVID19 previously.
  think_had_covid <-
    surv_df %>% filter(randsamp == "y") %>% 
    group_by(covid_illness) %>% count() %>%
    ## Modify counts so weights still reflect 
    ## King county population totals
    ungroup() %>% 
    mutate(Freq = round(n * sum(ps_obj$hisp_sex$Freq) / 
                          sum(n))) %>%
    select(-n)
  post_strat_obj <- survey::postStratify(
    ps_one_phase, ~covid_illness, think_had_covid
  )
  }else{
    post_strat_obj <- ps_one_phase
  }
  
  second_phase_one <- survey::svydesign(
    ids = ~HOUSEHOLD,
    weights = weights(post_strat_obj),
    strata = ~randsamp,
    data = subset(surv_df, qs == 1) 
  ) 
  
  post_rake_obj2 <- survey::rake(
    second_phase_one,
    sample.margins = list(
      ~AGECAT + sex,
      ~HISPANIC + sex,
      ~RACECAT + sex,
      ~INCOME + sex
      # ~INCOME + hhs
    ),
    population.margins = list(
      ps_obj$age_sex[, c("AGECAT", "sex", "Freq")],
      ps_obj$hisp_sex[, c("HISPANIC", "sex", "Freq")],
      ps_obj$race_sex[, c("RACECAT", "sex", "Freq")],
      ps_obj$inc_sex[, c("INCOME", "sex", "Freq")]
      # ps_obj$inc_hhs[, c("INCOME", "hhs", "Freq")]
    )
  )
  return(post_rake_obj2)
}

post_test <- make_ps_est(surv_df, post_cal_obj, type = "pos_test")
post_ill <- make_ps_est(surv_df, post_cal_obj, type = "cov_ill")
just_rake <- make_ps_est(surv_df, post_cal_obj, type = "none")
```

### 3.1.1 Percent Positive

```
make_table(list(#post_cal_obj,
                just_rake, post_test, post_ill), 
           c(#"Post-calibration two-phase object", 
             "Raked Object (main analysis)", 
             "Raked and balanced on positive tests", 
             "Raked and balanced on reporting illness"))
```

| Survey Object | Percent Positive (on both Tests) | |
| --- | --- | --- |
|  | Estimate | Standard Error |
| Raked Object (main analysis) | 3.24 | 0.73 |
| Raked and balanced on positive tests | 2.88 | 0.63 |
| Raked and balanced on reporting illness | 3.02 | 0.67 |

### 3.1.2 Prevalence Estimates

```
comp_prev_ests <- bind_rows(
  # post_adj_est(post_cal_obj), 
  post_adj_est(just_rake), 
  post_adj_est(post_test),
  post_adj_est(post_ill)
)

ess_cmpr <- sapply(list(just_rake, post_test, post_ill), 
       FUN = function(x) {
         round(
           nrow(x$variables) / 
             survey::svymean(~both_pos,x, deff = "replace") %>% 
             survey::deff())
       })

deffs_cmpr <- sapply(list(just_rake, post_test, post_ill), 
       FUN = function(x) {
             survey::svymean(~both_pos,x, deff = "replace") %>% 
             survey::deff() %>% round(2)
       })

comp_prev_ests$`Adjustment Strategy` <- 
  c(#"Post-calibration two-phase object", 
    "Raked Object (main analysis)", 
    "Raked and balanced on positive tests", 
    "Raked and balanced on reporting illness")
comp_prev_ests$`Design Effect` <- deffs_cmpr
comp_prev_ests$`Effective Sample Size` <- ess_cmpr
comp_prev_ests[, c(2, 3, 4, 1)] %>% gt::gt() %>% 
  gt::cols_align("center")
```

| Adjustment Strategy | Design Effect | Effective Sample Size | Prevalence Estimate (95% CI) |
| --- | --- | --- | --- |
| Raked Object (main analysis) | 2.18 | 589 | 3.91 (2.35, 5.96) |
| Raked and balanced on positive tests | 1.84 | 698 | 3.47 (2.17, 5.28) |
| Raked and balanced on reporting illness | 1.99 | 644 | 3.65 (2.15, 5.45) |

## 

### 3.1.3 Uncertainty from imputing question responses

When the above sensitivity analysis was conducted, individuals with missing values for answers to the COVID survey questions had their values imputed assuming missingness at random (conditional on strata). To study how this imputation effected the point estimates and standard error, here we carry out multiple imputations and study the distribution of the resulting estimate. Here balancing is carried out based on the distribution of individuals who reported feeling ill.

```
if (file.exists("mult_imp_sens.rds")) {
  all_res <- readRDS("mult_imp_sens.rds")
} else {
  all_res <- data.frame("est" = rep(NA, 1000), "se" = NA)
  
  for (res_idx in 1:nrow(all_res)) {
    cat(res_idx, " ")
    subobj <- make_ps_est(surv_df, post_cal_obj, type = "cov_ill")
    survey_res <- survey::svymean(~both_pos, subobj)
    all_res[res_idx, ] <- c("est" = as.numeric(survey_res),
                            "se" = survey_res %>% survey::SE())
  }
  saveRDS(all_res, file = "mult_imp_sens.rds")
}
```

```
## 1  2  3  4  5  6  7  8  9  10  11  12  13  14  15  16  17  18  19  20  21  22  23  24  25  26  27  28  29  30  31  32  33  34  35  36  37  38  39  40  41  42  43  44  45  46  47  48  49  50  51  52  53  54  55  56  57  58  59  60  61  62  63  64  65  66  67  68  69  70  71  72  73  74  75  76  77  78  79  80  81  82  83  84  85  86  87  88  89  90  91  92  93  94  95  96  97  98  99  100  101  102  103  104  105  106  107  108  109  110  111  112  113  114  115  116  117  118  119  120  121  122  123  124  125  126  127  128  129  130  131  132  133  134  135  136  137  138  139  140  141  142  143  144  145  146  147  148  149  150  151  152  153  154  155  156  157  158  159  160  161  162  163  164  165  166  167  168  169  170  171  172  173  174  175  176  177  178  179  180  181  182  183  184  185  186  187  188  189  190  191  192  193  194  195  196  197  198  199  200  201  202  203  204  205  206  207  208  209  210  211  212  213  214  215  216  217  218  219  220  221  222  223  224  225  226  227  228  229  230  231  232  233  234  235  236  237  238  239  240  241  242  243  244  245  246  247  248  249  250  251  252  253  254  255  256  257  258  259  260  261  262  263  264  265  266  267  268  269  270  271  272  273  274  275  276  277  278  279  280  281  282  283  284  285  286  287  288  289  290  291  292  293  294  295  296  297  298  299  300  301  302  303  304  305  306  307  308  309  310  311  312  313  314  315  316  317  318  319  320  321  322  323  324  325  326  327  328  329  330  331  332  333  334  335  336  337  338  339  340  341  342  343  344  345  346  347  348  349  350  351  352  353  354  355  356  357  358  359  360  361  362  363  364  365  366  367  368  369  370  371  372  373  374  375  376  377  378  379  380  381  382  383  384  385  386  387  388  389  390  391  392  393  394  395  396  397  398  399  400  401  402  403  404  405  406  407  408  409  410  411  412  413  414  415  416  417  418  419  420  421  422  423  424  425  426  427  428  429  430  431  432  433  434  435  436  437  438  439  440  441  442  443  444  445  446  447  448  449  450  451  452  453  454  455  456  457  458  459  460  461  462  463  464  465  466  467  468  469  470  471  472  473  474  475  476  477  478  479  480  481  482  483  484  485  486  487  488  489  490  491  492  493  494  495  496  497  498  499  500  501  502  503  504  505  506  507  508  509  510  511  512  513  514  515  516  517  518  519  520  521  522  523  524  525  526  527  528  529  530  531  532  533  534  535  536  537  538  539  540  541  542  543  544  545  546  547  548  549  550  551  552  553  554  555  556  557  558  559  560  561  562  563  564  565  566  567  568  569  570  571  572  573  574  575  576  577  578  579  580  581  582  583  584  585  586  587  588  589  590  591  592  593  594  595  596  597  598  599  600  601  602  603  604  605  606  607  608  609  610  611  612  613  614  615  616  617  618  619  620  621  622  623  624  625  626  627  628  629  630  631  632  633  634  635  636  637  638  639  640  641  642  643  644  645  646  647  648  649  650  651  652  653  654  655  656  657  658  659  660  661  662  663  664  665  666  667  668  669  670  671  672  673  674  675  676  677  678  679  680  681  682  683  684  685  686  687  688  689  690  691  692  693  694  695  696  697  698  699  700  701  702  703  704  705  706  707  708  709  710  711  712  713  714  715  716  717  718  719  720  721  722  723  724  725  726  727  728  729  730  731  732  733  734  735  736  737  738  739  740  741  742  743  744  745  746  747  748  749  750  751  752  753  754  755  756  757  758  759  760  761  762  763  764  765  766  767  768  769  770  771  772  773  774  775  776  777  778  779  780  781  782  783  784  785  786  787  788  789  790  791  792  793  794  795  796  797  798  799  800  801  802  803  804  805  806  807  808  809  810  811  812  813  814  815  816  817  818  819  820  821  822  823  824  825  826  827  828  829  830  831  832  833  834  835  836  837  838  839  840  841  842  843  844  845  846  847  848  849  850  851  852  853  854  855  856  857  858  859  860  861  862  863  864  865  866  867  868  869  870  871  872  873  874  875  876  877  878  879  880  881  882  883  884  885  886  887  888  889  890  891  892  893  894  895  896  897  898  899  900  901  902  903  904  905  906  907  908  909  910  911  912  913  914  915  916  917  918  919  920  921  922  923  924  925  926  927  928  929  930  931  932  933  934  935  936  937  938  939  940  941  942  943  944  945  946  947  948  949  950  951  952  953  954  955  956  957  958  959  960  961  962  963  964  965  966  967  968  969  970  971  972  973  974  975  976  977  978  979  980  981  982  983  984  985  986  987  988  989  990  991  992  993  994  995  996  997  998  999  1000
```

#### 3.1.3.1

```
hist(100 * all_res$est, xlab = "Percent positive estimate", main = "")
```

Here we see that while these imputations to introduce some variability in the percent positive estimate, this variability never results in an estimate that is much more than a tenth of a percent away from the original estimate.

#### 3.1.3.2

```
hist(100 * all_res$se, xlab = "Standad error", main = "")
```

Again we see that while these imputations have little effect on the estimated standard error of the percent positive estimate. Here the range of standard errors is less than five hundredths of a percent.

## 3.2 Variablility of point estimate from imperfect test accuracy measures

While the diagnostic tests do have reported sensitivities, and specificities, there is some uncertainty associated with these values. Here we seek to understand how much variability in our point estimate would be possible due to these factors. This is done using two approaches:

- Considering the four test accuracy measures (sens and spec for both tests), we choose values (either 5% or 95% quantiles) that would give the highest estimate of prevalence. Thus for both tests the 95% quantile for specificity is chosen (fewer individuals who tested positive are actually negative) and the 5% quantile for sensitivity (more individuals who tested negative are actually positive). These values are then used to calculate the sensitivity and specificity of the combined test, which is subsequently used to estimate the prevalence. An analogous method is used to obtain the low estimate of prevalence, but the small values for specificity and large values for sensitivity are used.
- The second approach uses a bootstrap to draw (independently) the four test performance measures and then uses the measures to calculate the performance of the combined test and the prevalence implied from this performance and the estimate of percentage of positive tests. These bootstraps use a normal distribution for sampling these measures. Taking the 5%, 50%, and 95% quantiles of this bootstrap distribution, we calculate the estimate and confidence intervals for these scenarios.

### 3.2.1 Choosing extreme values

```
shft <- qnorm(0.95) / qnorm(0.975)
lower_est <- function(x) {x[1] * (0.5 + shft) + x[2] * (0.5 - shft)}
upper_est <- function(x) {x[1] * (0.5 - shft) + x[2] * (0.5 + shft)}
lower_prev_scen <- lapply(test_info, FUN = function(x) {
  list("spec" = pmin(1, lower_est(x$spec)), 
       "sens" = pmin(1, upper_est(x$sens)))
})
upper_prev_scen <- lapply(test_info, FUN = function(x) {
  list("spec" = pmin(1, upper_est(x$spec)), 
       "sens" = pmin(1, lower_est(x$sens)))
} )

lw_p <- combine_tests(t1_spec = lower_prev_scen$first_test$spec,
                      t1_sens = lower_prev_scen$first_test$sens, 
                      t2_spec = lower_prev_scen$second_test$spec,
                      t2_sens = lower_prev_scen$second_test$sens) 

up_p <- combine_tests(t1_spec = upper_prev_scen$first_test$spec,
                      t1_sens = upper_prev_scen$first_test$sens, 
                      t2_spec = upper_prev_scen$second_test$spec,
                      t2_sens = upper_prev_scen$second_test$sens) 

look_three_scen <- function(low, mid, high, 
                            scen_names = c(
                             "Low Specificity, High Sensitivity",
                             "Best Guess Test Performance",
                             "High Specificity, Low Sensitivity"
                            )){
  mult_res <- bind_rows(
    bind_cols(
      Scenario = scen_names[1], 
      post_adj_est(post_rake_obj, se = low["sens"], sp = low["spec"])),
    bind_cols(Scenario = scen_names[2], 
              post_adj_est(post_rake_obj, se = mid["sens"], sp = mid["spec"])),
    bind_cols(Scenario = scen_names[3], 
              post_adj_est(post_rake_obj, 
                           se = high["sens"], sp = high["spec"])),
  )
  desn_eff <- survey::svymean(~both_pos, post_rake_obj, deff = "replace") %>% 
    survey::deff()
  mult_res %>% gt::gt() %>% gt::cols_align("center") %>% gt::tab_footnote(
    footnote = paste0("The effective sample size is ",
                      round(nrow(post_rake_obj$variables) / desn_eff), 
                      " for these calculations."),
    locations = gt::cells_column_labels(
      columns = vars(`Prevalence Estimate (95% CI)`))
  )
}
look_three_scen(lw_p, comb_sens_spec, up_p)
```

| Scenario | Prevalence Estimate (95% CI)1 |
| --- | --- |
| Low Specificity, High Sensitivity | 3.14 (1.85, 4.84) |
| Best Guess Test Performance | 3.91 (2.35, 5.96) |
| High Specificity, Low Sensitivity | 5.46 (3.29, 8.31) |
|  |  |
| --- | --- |
| *1* The effective sample size is 589 for these calculations. | |

### 3.2.2 Bootstrap distribution of combined test

```
# apply(many_sens_spec, 2, mean)
make_int <- function(test_positivity, sens_spec_df) {
  all_res <- matrix(NA, nrow = nrow(sens_spec_df), ncol = 3)
  for (b_idx in 1:nrow(all_res)) {
    all_res[b_idx, ] <-  c(adj_for_sesp(
      AP = test_positivity, 
      Sp = sens_spec_df[b_idx, "spec"],
      Se = sens_spec_df[b_idx, "sens"]),
      sens_spec_df[b_idx, "spec"],
      sens_spec_df[b_idx, "sens"]
    )
  }
  colnames(all_res) <- c("prev", "spec", "sens")
  return(all_res)
}

interval <- make_int(as.numeric(survey::svymean(~both_pos, post_rake_obj)), 
         many_sens_spec)

# hist(100 * interval[, 1], freq = FALSE, main = "Histogram of possible prevalence point estimates", xlab = "Prevalence Estimate (percentage)")
quants <- quantile(x = interval[, 1], c(0.05, 0.5, 0.95), type = 1)
new_df <- interval[match(quants, interval[, 1]), ]

look_three_scen(new_df[1, 2:3],  new_df[2, 2:3], new_df[3, 2:3], 
                c("Lower Prevalence", "Median Guess", "Higher Prevalence"))
```

| Scenario | Prevalence Estimate (95% CI)1 |
| --- | --- |
| Lower Prevalence | 3.78 (2.27, 5.78) |
| Median Guess | 4.00 (2.40, 6.11) |
| Higher Prevalence | 4.24 (2.54, 6.47) |
|  |  |
| --- | --- |
| *1* The effective sample size is 589 for these calculations. | |

# 

Above we see that the estimates vary less when considering the range of the combined test than when choosing extreme values for all four test accuracy measures.

# 4 Summary

After the many adjustments made, the estimated Seroprevalence estimate is:

```
fin_reslt
```

| Prevalence Estimate (95% CI)1 |
| --- |
| 3.91 (2.35, 5.96) |
|  |
| --- |
| *1* The effective sample size is 589 for these calculations. |

## 4.1 Limitations

While the above estimate provides a best guess at the prevalence in King county there are multiple limitations:

- The sample was constructed assuming that both a convenience sample and an address-based sample contribute equally to the analysis. This assumption is likely to have resulted in an estimate that is higher than the true prevalence. This is because individuals from the convenience sample were more likely to report being ill with COVID-like illness and more likely to have tested positive due to a COVID-like illness compared to the individuals in the address-based sample. A more careful construction of weights is possible using methods described in Valliant (2020), but would require discarding the weights that were already calculated.

  - A sensitivity analysis of this bias rebalanced the sample estimate based on questions regarding COVID19 illness. With this rebalancing the estimate of prevalence was roughly half a percent lower than the unbalanced estimate.
- In the calculations of weights, imputations were carried for individuals with missing values for any demographic covariates used to reweight the sample (age, sex, race, Hispanic status, and income). A hot-deck imputation method was used, except for Hispanic status in which all individuals with a missing value were assigned non-Hispanic. These weights were treated as fixed throughout the analysis. If the variability introduced by these imputations were included (by using a multiple imputation method for example) the confidence intervals for prevalence would likely be slightly wider.
- Two tests were used to confirm seropositivity for each individual. This analysis treated the two tests as a single test, by assuming that the tests performances were independent conditional on an individuals status. While there is little reason to think this assumption incorrect, if this assumption is incorrect the estimate of prevalence could be biased. Unfortunately checking this assumption is impossible with the current data because all individuals who tested negative on the initial test did not receive a second test.

# 5 Appendix: Percentage missingness

## 5.1 Crosstabs of Counts

```
# initial_df <- readxl::read_excel("../02_initial_resp_dat_preimp/initial_resp_dat.xlsx")
# imputed_df <- readxl::read_excel(
#   "../03_from_mansour_postimp/KC_Out_1.Weights from Mansour.xlsx",
#   sheet = 2)
# sub_inital <- match(unique(initial_df$hhid), initial_df$hhid)
# initial_df <- initial_df[sub_inital, ]
# 
# # here are all caps
# imputed_df$hhid <- paste0(imputed_df$HOUSEHOLD, "-", imputed_df$INDIVIDUAL)
# sub_imputed <- match(unique(imputed_df$hhid), imputed_df$hhid)
# imputed_df <- imputed_df[sub_imputed, ]
# 
# cmbd_dfs <- left_join(imputed_df, initial_df, by = "hhid")
# 
# cmbd_dfs$pre_imp_agecat <- agrp_nms[cut(cmbd_dfs$ageyrs, agrp_lvs,
#                                include.lowest = TRUE, labels = FALSE)]
# cmbd_dfs$INCOME <- cmbd_dfs$INCOME - 1
```

Below are given crosstabs of the values pre- and post-imputation for each variable used for the construction of sampling weights. Rows correspond to the post-imputation values and columns corresponded to the pre-imputation values.

### 5.1.1 Sex

```
## Compare Sex
surv_df$sex <- c("M", "F")[surv_df$sex]
knitr::kable(table(surv_df[, c("sex", "sex")], useNA = "al"))
```

|  | F | M | NA |
| --- | --- | --- | --- |
| F | 714 | 0 | 0 |
| M | 0 | 650 | 0 |
| NA | 0 | 0 | 0 |

### 5.1.2 Race Category

```
knitr::kable(table(surv_df[, c("RACECAT", "race1")], useNA = "al"))
```

|  | A | AW | B | N | NW | P | R | U | W | NA |
| --- | --- | --- | --- | --- | --- | --- | --- | --- | --- | --- |
| 1 | 0 | 2 | 0 | 0 | 1 | 0 | 77 | 31 | 840 | 2 |
| 2 | 0 | 0 | 76 | 0 | 0 | 0 | 5 | 1 | 0 | 0 |
| 3 | 254 | 0 | 0 | 0 | 0 | 0 | 15 | 9 | 0 | 1 |
| 4 | 0 | 0 | 0 | 23 | 0 | 18 | 8 | 1 | 0 | 0 |
| NA | 0 | 0 | 0 | 0 | 0 | 0 | 0 | 0 | 0 | 0 |

### 5.1.3 Race Category (refactored)

```
level_key <- c("1" = "White", "2" = "Black", "3" = "Asian", "4" = "Other")
surv_df$RACECAT_with_NA <- recode(
  surv_df$race1, .default = NA_real_, .missing = NA_real_, 
  A = 3, AW = 1, B = 2, N = 4, P = 4, NW = 1, 
  W = 1, R = NA_real_, U = NA_real_,
)

surv_df$RACECAT <- recode(surv_df$RACECAT, !!!level_key)
surv_df$RACECAT_with_NA <- recode(surv_df$RACECAT_with_NA, !!!level_key)
knitr::kable(table(surv_df[, c("RACECAT", "RACECAT_with_NA")], useNA = "al"))
```

|  | Asian | Black | Other | White | NA |
| --- | --- | --- | --- | --- | --- |
| Asian | 254 | 0 | 0 | 0 | 25 |
| Black | 0 | 76 | 0 | 0 | 6 |
| Other | 0 | 0 | 41 | 0 | 9 |
| White | 0 | 0 | 0 | 843 | 110 |
| NA | 0 | 0 | 0 | 0 | 0 |

### 5.1.4 Income Level

```
inc_key <- c("< $20,000", "$20,000 - $40,000", "$40,001 - $60,000",
             "$60,001 - $80,000", "$80,001 - $100,000", 
             "$100,001 - $120,000", "$120,001+")
names(inc_key) <- 1:7
surv_df$INCOME <- factor(x = surv_df$INCOME, levels = 1:7,
                          labels = inc_key)
surv_df$INCOME_with_NA <- surv_df$INCOME
surv_df$INCOME_with_NA[which(is.na(surv_df$binary_income_with_NA))] <- NA
knitr::kable(table(surv_df[, c("INCOME", "INCOME_with_NA")], useNA = "al"))
```

|  | < $20,000 | $20,000 - $40,000 | $40,001 - $60,000 | $60,001 - $80,000 | $80,001 - $100,000 | $100,001 - $120,000 | $120,001+ | NA |
| --- | --- | --- | --- | --- | --- | --- | --- | --- |
| < $20,000 | 50 | 0 | 0 | 0 | 0 | 0 | 0 | 6 |
| $20,000 - $40,000 | 0 | 101 | 0 | 0 | 0 | 0 | 0 | 10 |
| $40,001 - $60,000 | 0 | 0 | 113 | 0 | 0 | 0 | 0 | 8 |
| $60,001 - $80,000 | 0 | 0 | 0 | 135 | 0 | 0 | 0 | 18 |
| $80,001 - $100,000 | 0 | 0 | 0 | 0 | 129 | 0 | 0 | 15 |
| $100,001 - $120,000 | 0 | 0 | 0 | 0 | 0 | 141 | 0 | 16 |
| $120,001+ | 0 | 0 | 0 | 0 | 0 | 0 | 571 | 51 |
| NA | 0 | 0 | 0 | 0 | 0 | 0 | 0 | 0 |

### 5.1.5 Age Group

```
agrp_lvs <- c(0, 12, 17, 24, 34, 44, 64,100)
agrp_nms <- c("0≤age≤12",
              paste0(agrp_lvs[-c(1, length(agrp_lvs))],
                     "<age≤",
                     agrp_lvs[-(1:2)]))
surv_df$AGECAT <- agrp_nms[surv_df$AGECAT]
surv_df$pre_imp_agecat <- surv_df$AGECAT
surv_df$pre_imp_agecat[which(is.na(surv_df$agegrp2_with_NA))] <- NA
knitr::kable(table(surv_df[, c("AGECAT", "pre_imp_agecat")], useNA = "al"))
```

|  | 0≤age≤12 | 12<age≤17 | 17<age≤24 | 24<age≤34 | 34<age≤44 | 44<age≤64 | 64<age≤100 | NA |
| --- | --- | --- | --- | --- | --- | --- | --- | --- |
| 0≤age≤12 | 127 | 0 | 0 | 0 | 0 | 0 | 0 | 7 |
| 12<age≤17 | 0 | 62 | 0 | 0 | 0 | 0 | 0 | 4 |
| 17<age≤24 | 0 | 0 | 77 | 0 | 0 | 0 | 0 | 2 |
| 24<age≤34 | 0 | 0 | 0 | 213 | 0 | 0 | 0 | 6 |
| 34<age≤44 | 0 | 0 | 0 | 0 | 263 | 0 | 0 | 6 |
| 44<age≤64 | 0 | 0 | 0 | 0 | 0 | 381 | 0 | 10 |
| 64<age≤100 | 0 | 0 | 0 | 0 | 0 | 0 | 198 | 8 |
| NA | 0 | 0 | 0 | 0 | 0 | 0 | 0 | 0 |

### 5.1.6 Hispanic Ethnicity

```
knitr::kable(table(surv_df[, c("HISPANIC", "HISPANIC_with_NA")], useNA = "al"))
```

|  | 1 | 2 | NA |
| --- | --- | --- | --- |
| 1 | 98 | 0 | 0 |
| 2 | 0 | 645 | 621 |
| NA | 0 | 0 | 0 |

## 5.2 Percentage of missing values for each covariate

```
prc_ms <- function(x) round(mean(is.na(x)) * 100, 2)
percent_missing <- apply(
  surv_df[, c("sex", "pre_imp_agecat", "INCOME_with_NA", "RACECAT_with_NA",
               "HISPANIC_with_NA")], 
      2, prc_ms)

fin_df <- tibble("Variable" = c("Sex", "Age Category", "Income", 
                                    "Race", "Ethnicity"), 
                     "Percent Imputed" = percent_missing)
gt::gt(fin_df)
```

| Variable | Percent Imputed |
| --- | --- |
| Sex | 0.00 |
| Age Category | 3.15 |
| Income | 9.09 |
| Race | 11.00 |
| Ethnicity | 45.53 |

```
make_table <- function(impt_col, orig_col, my_df = surv_df, cnm = NULL){
  RACECAT <- enexpr(impt_col) 
  col_race <- enexpr(orig_col)
  race_sum <- my_df %>% group_by(!!RACECAT) %>% 
    summarise("n" = n(),
              "Count Imputed" = sum(is.na(!!col_race)),
              "Percent Imputed" = prc_ms(!!col_race)) %>% 
    ungroup() %>%
    mutate("Percentage of Sample" = round(100 * n/sum(n), 2)) %>%
    rename("Count All" = n)
  tots <- race_sum %>% summarise(
    name = "Total",
    "Count All" = sum(`Count All`),
    "Count Imputed" = sum(`Count Imputed`), 
    "Percent Imputed" = round(
      100 *  sum(`Count Imputed`) / sum(`Count All`), 2), 
    "Percentage of Sample" = 100) 
  colnames(tots)[1] <- colnames(race_sum)[1] 
  all_inf <- rbind(race_sum, tots)
  all_inf <- all_inf[, c(1, 2, 5, 3, 4)]
  if (!is.null(cnm)) {colnames(all_inf)[1] <- cnm}
  gt::gt(all_inf) %>%
    gt::tab_style(
    style = list(
      gt::cell_text(weight = "bold")
      ),
    locations = gt::cells_body(
        rows = nrow(all_inf)
  )
    )
}
```

## 5.3 Percentage of final values that were imputed:

These tables show the per-covariate proportion of values that were imputed. As an example, if 90 individuals in the original sample indicated being between 17 and 24 years old, but after imputation there are 100 individuals who are classified as being between 17 and 24 years old, then the percentage of final values that were imputed would be \((100 - 90) / 100 = 10\%\).

### 5.3.1 Sex

```
make_table(sex, sex, cnm = "Sex")
```

| Sex | Count All | Percentage of Sample | Count Imputed | Percent Imputed |
| --- | --- | --- | --- | --- |
| F | 714 | 52.35 | 0 | 0 |
| M | 650 | 47.65 | 0 | 0 |
| Total | 1364 | 100.00 | 0 | 0 |

### 5.3.2 Race

```
make_table(RACECAT, RACECAT_with_NA, cnm = "Race")
```

| Race | Count All | Percentage of Sample | Count Imputed | Percent Imputed |
| --- | --- | --- | --- | --- |
| Asian | 279 | 20.45 | 25 | 8.96 |
| Black | 82 | 6.01 | 6 | 7.32 |
| Other | 50 | 3.67 | 9 | 18.00 |
| White | 953 | 69.87 | 110 | 11.54 |
| Total | 1364 | 100.00 | 150 | 11.00 |

### 5.3.3 Income Level

These are the income levels used to create weights for the analysis.

```
make_table(INCOME, INCOME_with_NA, cnm = "Income")
```

| Income | Count All | Percentage of Sample | Count Imputed | Percent Imputed |
| --- | --- | --- | --- | --- |
| < $20,000 | 56 | 4.11 | 6 | 10.71 |
| $20,000 - $40,000 | 111 | 8.14 | 10 | 9.01 |
| $40,001 - $60,000 | 121 | 8.87 | 8 | 6.61 |
| $60,001 - $80,000 | 153 | 11.22 | 18 | 11.76 |
| $80,001 - $100,000 | 144 | 10.56 | 15 | 10.42 |
| $100,001 - $120,000 | 157 | 11.51 | 16 | 10.19 |
| $120,001+ | 622 | 45.60 | 51 | 8.20 |
| Total | 1364 | 100.00 | 124 | 9.09 |

### 5.3.4 Binary Income Level

These are the income levels used for the subgroup analysis.

```
make_table(binary_income, binary_income_with_NA, surv_df, "Binary Income")
```

| Binary Income | Count All | Percentage of Sample | Count Imputed | Percent Imputed |
| --- | --- | --- | --- | --- |
| Above 100,000 | 779 | 57.11 | 67 | 8.60 |
| At or below 100,000 | 585 | 42.89 | 57 | 9.74 |
| Total | 1364 | 100.00 | 124 | 9.09 |

### 5.3.5 Age Group

These are the age groups used to create weights for the analysis.

```
make_table(AGECAT, pre_imp_agecat, cnm = "Age Group")
```

| Age Group | Count All | Percentage of Sample | Count Imputed | Percent Imputed |
| --- | --- | --- | --- | --- |
| 0≤age≤12 | 134 | 9.82 | 7 | 5.22 |
| 12<age≤17 | 66 | 4.84 | 4 | 6.06 |
| 17<age≤24 | 79 | 5.79 | 2 | 2.53 |
| 24<age≤34 | 219 | 16.06 | 6 | 2.74 |
| 34<age≤44 | 269 | 19.72 | 6 | 2.23 |
| 44<age≤64 | 391 | 28.67 | 10 | 2.56 |
| 64<age≤100 | 206 | 15.10 | 8 | 3.88 |
| Total | 1364 | 100.00 | 43 | 3.15 |

### 5.3.6 Other Age Group

These are the age groups used for the subgroup analysis.

```
make_table(agegrp2, agegrp2_with_NA, surv_df, "Age Group")
```

| Age Group | Count All | Percentage of Sample | Count Imputed | Percent Imputed |
| --- | --- | --- | --- | --- |
| 0≤age<16 | 171 | 12.54 | 9 | 5.26 |
| 16≤age<25 | 108 | 7.92 | 4 | 3.70 |
| 25≤age<45 | 488 | 35.78 | 12 | 2.46 |
| 45≤age<65 | 391 | 28.67 | 10 | 2.56 |
| 65≤age<100 | 206 | 15.10 | 8 | 3.88 |
| Total | 1364 | 100.00 | 43 | 3.15 |

### 5.3.7 Ethnicicty

```
make_table(HISPANIC, HISPANIC_with_NA, cnm = "Ethnicity")
```

| Ethnicity | Count All | Percentage of Sample | Count Imputed | Percent Imputed |
| --- | --- | --- | --- | --- |
| 1 | 98 | 7.18 | 0 | 0.00 |
| 2 | 1266 | 92.82 | 621 | 49.05 |
| Total | 1364 | 100.00 | 621 | 45.53 |

# Sources

“Anti-SARS-CoV-2 ELISA (IgG) Instruction for Use.” n.d. EUROIMMUN. https://www.fda.gov/media/137609/download.

“Epitools - Sensitivity and Specificity of Two Tests Used ...” n.d. Accessed February 24, 2021. https://epitools.ausvet.com.au/twoteststwo.

Lumley, Thomas. 2010. *Complex Surveys: A Guide to Analysis Using R*. 1st edition. Hoboken, N.J: Wiley.

———. 2020. “Survey: Analysis of Complex Survey Samples.” https://CRAN.R-project.org/package=survey.

“Platelia SARS-CoV-2 Total Ab.” n.d. Bio-Rad. https://www.bio-rad.com/webroot/web/pdf/cdg/literature/IFU\_72710\_12013798\_16008597\_2020\_06\_EN\_US.pdf.

REICZIGEL, J., J. FÖLDI, and L. ÓZSVÁRI. 2010. “Exact Confidence Limits for Prevalence of a Disease with an Imperfect Diagnostic Test.” *Epidemiology and Infection* 138 (11): 1674–78. https://www.jstor.org/stable/40928502.

Valliant, Richard. 2020. “Comparing Alternatives for Estimation from Nonprobability Samples.” *Journal of Survey Statistics and Methodology* 8 (2): 231–63. https://doi.org/10.1093/jssam/smz003.
